# Omics Insights Into the Effects of Highbush Blueberry and Cranberry Crop Agroecosystems on Honey Bee Health and Physiology

**DOI:** 10.1002/pmic.70033

**Published:** 2025-09-06

**Authors:** Huan Zhong, Yuming Shi, Aleksandra Kozlova, Renata Moravcova, Jason C. Rogalski, Aidan Jamieson, Lance Lansing, Kyung‐Mee Moon, Xiaojing Yuan, Amanda S. Gregoris, Heather Higo, Julia Common, Ida M. Conflitti, Mateus Pepinelli, Lan Tran, Morgan Cunningham, Hosna Jabbari, Syed Abbas Bukhari, Sarah K. French, Rodrigo Ortega Polo, Shelley E. Hoover, Stephen F. Pernal, Pierre Giovenazzo, M. Marta Guarna, Amro Zayed, Leonard J. Foster

**Affiliations:** ^1^ Department of Biochemistry and Molecular Biology Michael Smith Laboratories Life Sciences Institute University of British Columbia Vancouver British Columbia Canada; ^2^ National Research University Higher School of Economics Moscow Russian Federation; ^3^ Proteomics and Metabolomics Core Facility Life Sciences Institute University of British Columbia Vancouver British Columbia Canada; ^4^ Department of Biology York University Toronto Ontario Canada; ^5^ Agriculture and Agri‐Food Canada Lethbridge Research and Development Centre Lethbridge Alberta Canada; ^6^ Agriculture and Agri‐Food Canada Beaverlodge Research Farm Beaverlodge Alberta Canada; ^7^ School of Natural Sciences Laurentian University Sudbury Ontario Canada; ^8^ Department of Computer Science University of Victoria Victoria British Columbia Canada; ^9^ Department of Biomedical Engineering University of Alberta Edmonton Alberta Canada; ^10^ Department of Biological Sciences University of Lethbridge Lethbridge Alberta Canada; ^11^ Département de Biologie Université Laval Ville de Québec Québec Canada; ^12^ Project Apis m Salt Lake City Utah USA; ^13^ Department of Biochemistry & Molecular Biology University of British Columbia Vancouver British Columbia Canada

**Keywords:** *Apis mellifera*, microbiome, multi‐omics, proteomics, transcriptomics

## Abstract

Honey bees (*Apis mellifera*) are vital pollinators in fruit‐producing agroecosystems like highbush blueberry (HBB) and cranberry (CRA). However, their health is threatened by multiple interacting stressors, including pesticides, pathogens, and nutritional changes. We tested the hypothesis that distinct agricultural ecosystems—with different combinations of agrochemical exposure, pathogen loads, and floral resources—elicit ecosystem‐specific, tissue‐level molecular responses in honey bees. We conducted an integrated multi‐omics analysis using RNA‐sequencing (RNA‐seq), proteomics, and gut microbiome profiling across three key tissue types (head, abdomen, and gut) of honey bees collected from two agroecosystems over two field seasons. Quantification was performed for pesticide residues, pathogen loads (*Nosema* spp., *Varroa destructor*, and multiple viruses), and gut microbiota. Weighted gene co‐expression network analysis (WGCNA) revealed tissue‐specific protein modules with ecosystem‐associated patterns, which differed from RNA co‐expression networks. Microbiome composition also varied, with key genera like *Gilliamella, Snodgrassella, and Bartonella* correlating with metabolic modules. These findings underscore the complex, environment‐dependent impacts of agroecosystem conditions on bee health. Our study provides a system‐level understanding of how combined pesticide, pathogen, and parasitic stressors, mediated by diet and microbiome, shape molecular phenotypes in honey bees—informing strategies for pollinator protection in managed landscapes.

**Summary:**

- This study provides a comprehensive multi‐omics analysis of honey bees foraging in blueberry and cranberry agroecosystems, offering novel insights into the molecular mechanisms underlying pollinator health in managed crop environments.
- By integrating transcriptomic, proteomic, and microbiome profiling across key tissues—head, abdomen, and gut—we reveal how environmental stressors, including pesticide exposure, pathogen infections, and parasitic infestations (e.g., *Varroa destructor*), differentially impact bee physiology and microbiome composition.
- Our findings highlight tissue‐specific responses to these stressors, with distinct metabolic pathway alterations observed in each tissue.
- Proteomic and transcriptomic analyses uncovered dysregulated pathways linked to oxidative phosphorylation and protein synthesis, while microbiome analysis revealed crop‐dependent shifts in gut bacterial communities, suggesting potential roles in pesticide detoxification and immune modulation.
- Notably, we identified key molecular biomarkers associated with stress adaptation, which may serve as early indicators of colony health deterioration.
- This research underscores the need for a system‐level approach to understanding pollinator stress in agricultural landscapes.
- By elucidating the interactions between diet, pesticide residues, pathogen loads, and molecular stress responses, our study provides a foundation for targeted conservation strategies aimed at mitigating environmental risks and improving pollination sustainability in agroecosystems.

**Abbreviations**
A205: absorbance at 205 nm
ACN: acetonitrile
BAM: binary alignment map
BC: British Columbia
BCA: bicinchoninic acid
BH: Benjamini‐Hochberg
BP: biological processes
BQCV: black queen cell virus
CAA: chloroacetamide
CC: cellular components
COLOSS: prevention of honey bee COlony LOSSes
CRA: cranberry
CSI: cold‐spray ionization
DDA: data‐dependent acquisition
DEGs: differentially expressed genes
DEPs: differentially expressed proteins
DIA: data‐independent acquisition
DTT: dithiothreitol
DWV: deformed wing virus
DWV‐A: deformed wing virus types A
DWV‐B: deformed wing virus types B
FDR: false discovery rate
GO: gene ontology
HBB: highbush blueberry
HPLC: high‐performance liquid chromatography
IAPV: Israeli acute paralysis virus
IEC: International Electrotechnical Commission
ISO: International Organization for Standardization
KEGG: Kyoto Encyclopedia of Genes and Genomes
lncRNA: long non‐coding RNA
MassIVE: Mass Spectrometry Interactive Virtual Environment
MF: molecular functions
MS/MS: tandem mass spectrometry
NCBI: National Center for Biotechnology Information
ncRNA: non‐coding RNA
OIE: Office International des Epizooties
PAGE: polyacrylamide gel electrophoresis
PASEF: parallel accumulation‐serial fragmentation
PCoA: principal coordinate analysis
PSM: peptide‐spectrum matches
qPCR: quantitative polymerase chain reaction
RNA‐seq: RNA sequencing
rRNA: ribosomal RNA
SBV: sacbrood virus
SDS: sodium dodecyl sulfate
snRNA: small nuclear RNA
sRNA: small RNA
TFA: trifluoroacetic acid
timsTOF: trapped ion mobility spectrometry time‐of‐flight
Tris: tris(hydroxymethyl)aminomethane
UHPLC: ultra‐high performance liquid chromatography
UV: ultraviolet
WGCNA: weighted gene coexpression network analysis

## 1 Introduction

Honey bees (*Apis mellifera*) play a crucial role in maintaining ecosystem stability and agricultural productivity by serving as primary pollinators for a diverse range of plant species. Their pollination services sustain biodiversity, facilitate plant reproduction, and contribute to global food security [1, 2]. Among economically significant crops, blueberries (*Vaccinium* spp.) and cranberries (*Vaccinium macrocarpon*) are particularly dependent on honey bee pollination [3, 4], as their fruit set, yield, and quality are directly influenced by pollinator activity [5, 6]. In British Columbia (BC), highbush blueberry (HBB) and cranberry (CRA) crops are cultivated in overlapping landscapes—particularly in the Fraser Valley—but exhibit distinct bloom periods, typically separated by five weeks. This temporal separation allows assessment of ecosystem‐level exposures without direct crop comparisons. Moreover, recent studies [7] have shown that BC blueberry fields are frequently pollinator‐limited, with insufficient bee activity constraining yield [8]. Similarly, in Québec, cranberry fields also depend heavily on managed pollination services [9, 10]. These systems, therefore, represent agriculturally intensive, pollination‐dependent environments that are ideal for studying how exposure to real‐world agroecosystem stressors affects honey bee physiology.

Given the increasing reliance on managed pollination services for these crops, ensuring honey bee health is essential for both ecological and agricultural sustainability [8]. Despite their ecological and economic importance, honey bees face increasing physiological and ecological stress due to a complex interplay of environmental factors, including habitat loss, climate change, pesticide exposure, and pathogen infections [11]. While global stocks of managed honey bees are increasing in number, particularly due to commercial beekeeping, this growth has not kept pace with the rising demand for pollination services in agriculture [12]. Moreover, colony health and survival remain under pressure, with high annual losses reported in many regions [13]. Agricultural intensification and monoculture practices have reduced floral diversity, leading to nutritional deficiencies in bees [14]. Simultaneously, widespread pesticide application has introduced chronic chemical exposure affecting colony health and profitability [15], while globalization has facilitated the spread of parasites and infectious diseases such as *V. destructor* and *V. destructor*‐vectored viruses [16, 17]. The combination of these stressors has been linked to increased colony losses and a growing mismatch between the rising demand for pollination and the availability of healthy pollinator populations [18]. Despite extensive research, a critical gap remains in understanding how these stressors interact at the molecular and tissue‐specific levels.

Honey bee physiology is highly compartmentalized, with different tissues performing distinct biological functions. The head governs neural processing and sensory perception [19], the thorax houses the muscles responsible for locomotion and flight [20, 21], while the abdomen regulates metabolic and immune responses [22]. The gut, located within the abdomen, plays a key role in digestion, detoxification, and microbiome interactions [23]. Studies have shown that stressors such as pesticides and pathogens exert distinct effects on different tissues, suggesting that whole‐body analyses may overlook important physiological responses [24]. For instance, chronic pesticide exposure has been associated with neurological impairments in the head, immune suppression in the abdomen, and microbiome dysbiosis in the gut [25]. Despite these insights, integrated, tissue‐specific investigations into the molecular mechanisms underlying stressor interactions remain limited.

Recent advances in multi‐omics technologies, including transcriptomics, proteomics, and microbiome profiling, offer powerful tools for unraveling the molecular basis of stress responses in honey bees. Transcriptomic analysis provides insights into changes in gene expression patterns in response to environmental stressors [26, 27], proteomics identifies alterations in protein abundance and function related to stress adaptation [28], and metagenomics reveals shifts in gut microbial composition that may influence detoxification, digestion, and immune responses in honey bees and other agricultural organisms [29, 30]. While omics‐based studies have enhanced our understanding of honey bee physiology, integrative, tissue‐specific multi‐omics studies remain scarce, making it difficult to comprehensively assess how different stressors interact across biological layers.

Among the most significant environmental threats to honey bees are pesticides and pathogens, which often act synergistically to exacerbate physiological stress [31]. Neonicotinoids, fungicides, and insect growth regulators have been shown to impair detoxification mechanisms, disrupt neurological function, and alter immune pathways [32]. Additionally, pesticide exposure has been linked to increased susceptibility to pathogens such as *Nosema* spp. and deformed wing virus (DWV), leading to higher mortality rates and reduced colony viability [25]. Experimental studies have demonstrated that pesticide exposure can compromise immune defenses, making bees more vulnerable to viral replication and parasitic infections [33]. Despite this evidence, the molecular mechanisms underlying pesticide‐pathogen interactions remain poorly characterized, particularly at the tissue level.

This study integrates 2 years of field‐collected data with multi‐omics profiling of the head, abdomen, and gut of honey bees exposed to pesticides and pathogens in highbush blueberry and cranberry pollination environments. We characterize tissue‐specific molecular responses, assess potential synergistic effects of these stressors on honey bee physiology, and identify key biological pathways underlying resilience and vulnerability. We are utilizing the Weighted Gene Co‐Expression Network Analysis (WGCNA) [34] to construct the protein co‐expression networks as well as the transcriptome co‐expression networks in order to reduce the feature dimensionality to seven key modules. By linking molecular data with real‐world exposure conditions, this study offers a system‐level perspective on honey bee stress responses. These insights can help guide pollinator conservation strategies and sustainable agricultural practices while informing targeted interventions to mitigate environmental stressors affecting bee populations.

## 2 Materials and Methods

### 2.1 Sample Collection and Processing

**FIGURE 1:**
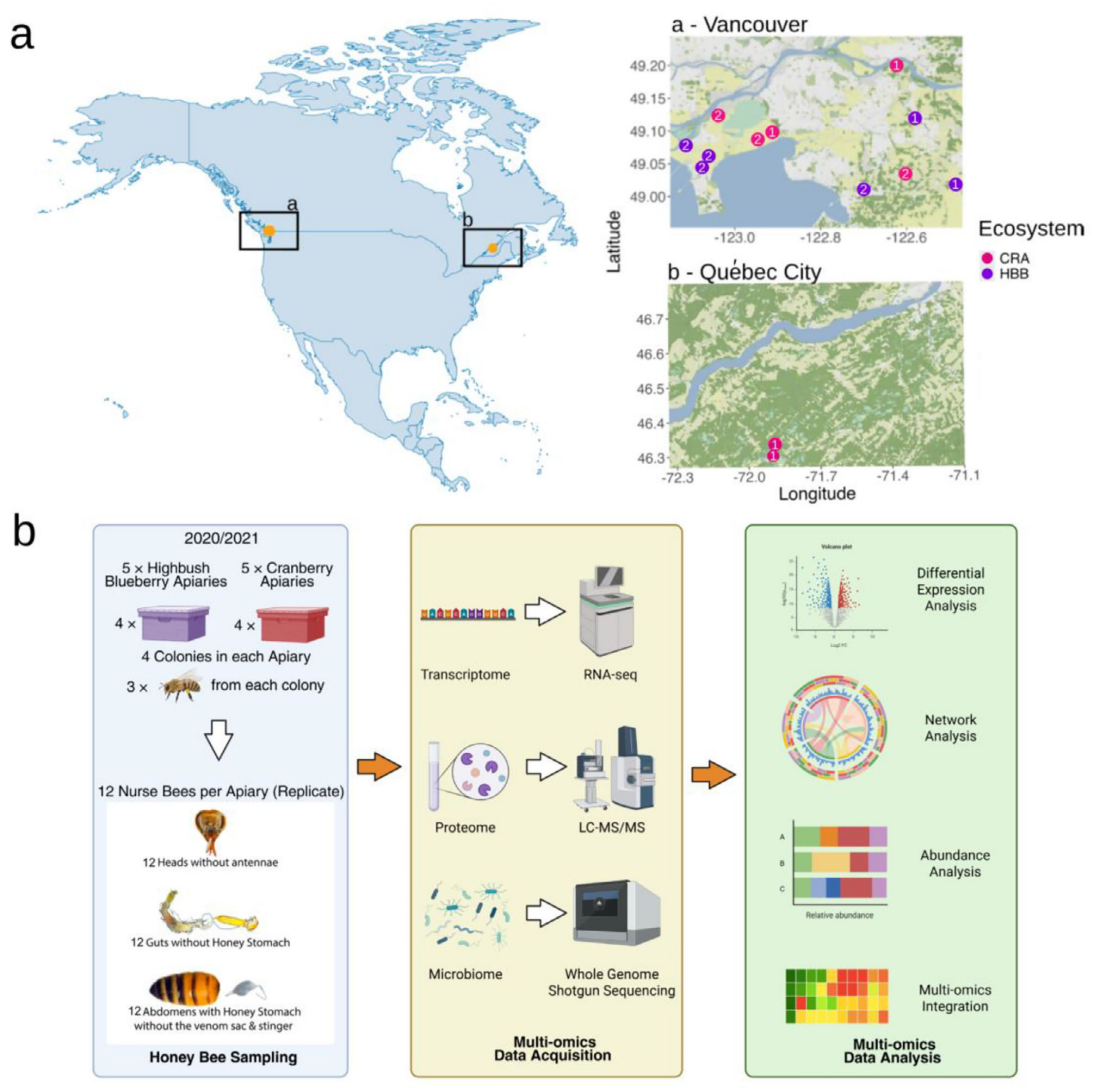
Experimental design and multi‐omics workflow for honey bee sampling across agroecosystems. (a) Sampling sites near Vancouver, BC and Québec City, Québec, the number of samples collected from each site is labelled on each dot, sampling details are available in Figure S1. (b) Nurse bees were collected in 2020 and 2021 from colonies located in highbush blueberry and cranberry fields. LC‐MS/MS was applied to obtain proteomes, RNA sequencing for transcriptomics and DNA metagenomic sequencing for microbiome profiling as described in materials and methods. Multi‐omics integration and biomarkers and functional analysis were performed. The figure was created in Biorender (https://BioRender.com/3k3xj93).

Field sampling was conducted to evaluate the molecular and physiological responses of honey bees in two types of ecosystems. The study investigated apiaries across 10 sites in 2020 and another 10 sites in 2021 (locations were shown in Figure 1 and Figure S1, and some sites were sampled in both years). Each site was considered an apiary replicate, with five near blueberry fields and five near cranberry fields. These apiaries were labeled “near” highbush blueberries or cranberries in previous studies [5, 35]. During the pollination period, samples were collected at the peak of blueberry bloom from colonies located near the blueberry fields [36].

There were four colonies at each apiary, and three nurse bees were collected from each colony. Nurse bees from each apiary were dissected into three tissue categories for proteomics and transcriptomics: the head (antennae removed), the abdomen (gut and stinger removed), and the midgut. For microbiome analysis, the entire digestive system (gut), excluding the honey crop, was dissected. Within each replicate apiary and tissue type, we pooled dissections from 12 individuals to generate a single sample for each tissue and apiary replicate. These pooled samples were used for proteomics analysis (*n* = 60), transcriptome analysis (*n* = 60), and for microbiome profiling (using only whole gut samples, *n* = 20) (Figure 1).

### 2.2 Transcriptome

The pooled samples were then homogenized with ceramic beads in a Fisherbrand Bead Mill 24 (Thermo Fisher) according to the RNeasy (Qiagen) manual. RNA was then extracted with the KingFisher Flex system according to the NucleoMag RNA kit (Thermo Fisher). Purified RNA samples were sent to Genome Québec (Montreal, Québec) for library preparation and paired‐end sequencing on a NovaSeq 6000 (Illumina) with an average depth of 50 million reads. The reads were aligned to the current honey bee genome (Amel HAv3.1 [37]) using STAR v2.9.7a [38] with default parameters. Afterwards, the resulting BAM files were used to generate count matrices via HTSeq‐Count v0.13.5 [39] with parameters: “non‐strandedness” and the feature counting mode set to “intersection‐nonempty”. The count files were used to perform differential expression analysis with edgeR [40]. Differentially expressed genes (DEGs) were determined from the model if they had an adjusted *p* value (false discovery rate (FDR) using a Benjamini‐Hochberg (BH) correction [41]) less than 0.05.

### 2.3 Proteome

#### 2.3.1 Library Generation

To generate the spectral library for data‐independent acquisition (DIA) proteomics, we created a pooled protein sample by randomly selecting three bee samples across a representative combination of ecosystems, tissues, sites, and years. Specifically, three samples were randomly chosen from our overall test pool to capture general protein representation across tissue types. The dissected tissues were lysed, homogenized, and quantified as described below. Briefly, dissected tissues were lysed in lysis buffer (4% SDS, 100 mM Tris pH 6.8, 1X protease inhibitors—Thermo Scientific Halt Protease Inhibitor Cocktail & cOmplete Protease Inhibitor Cocktail), homogenized in a Precellys 24 bead mill with 2.8 mm ceramic beads using three cycles of 30 s at 6.5 m/s, and followed by BCA protein quantification [42].

Next, 600 µg of the pooled protein sample of head, abdomen and 720 µg from the gut were used for further processing. Samples were reconstituted in 5 mM NH_4_HCO_2_, 2% ACN, pH 10, for HPLC separation of 50 µg in total on‑column. The digest was separated using Agilent 1100 LC series (Agilent) with InfinityLab Poroshell HPH‐C18 (2.1 × 100 mm, 4 µm) narrow bore LC column (Agilent) coupled to a UV detector. Two kinds of buffer were used in the following process: buffer A consisted of 5 mM NH_4_HCO_2_, 2% ACN in water, pH 10, and buffer B consisted of 5 mM NH_4_HCO_2_, 90% ACN. A standard 80‐minute run was performed with the following gradient: 0% to 13% solvent B over the first 30 min, then increased to 40% B from 30 to 60 min, followed by a ramp to 90% B over 2 min. The gradient was held at 90% B for 6 min, then increased to 100% B over 1 min and held for the final 11 min. Before each run, the analytical column was conditioned with 4 µL of buffer A. The Agilent thermostat temperature was maintained at 6°C. The flow rate was set to 100 µL/min. Fraction collection was triggered in time‐based mode with 1‐min slices. Fractions were dried down after collection [43, 44, 45]. The concatenated fractions were separated using a NanoElute UHPLC system (Bruker Daltonics) coupled to timsTOF Pro (Bruker Daltonics) operated in DDA‐PASEF mode, with parameters as previously described in Kolic et al. [46]

#### 2.3.2 Experimental Samples

Samples were processed, and proteins were extracted and measured as described above. Next, 40 µg of lysate from head and abdomen and 60 µg from gut lysed sample were taken for further processing. Reduction of disulfide bonds was done by incubation with 30 mM dithiothreitol (DTT) for 30 min at 37°C, followed by alkylation with 50 mM chloroacetamide (CAA) for 20 min at 37°C in the dark. Samples were then protein purified by SDS‐PAGE (10% Mini‐PROTEAN TGX Precast Protein Gels, Biorad [47]), and in‐gel digested with a total of 0.45 µg of trypsin (Promega) at 37°C [48]. Peptides were cleaned up via STAGE‐Tip purification [49]. Each of the samples was forced through a conditioned and equilibrated homemade column with 11 mm of C18 packing, washed with 0.1% TFA twice, and eluted into clean tubes by a buffer containing 40% ACN and 0.1% TFA, then dried down.

As described in [46], before LC‐MS/MS analysis, each sample was reconstituted in 0.5% ACN and 0.1% formic acid. Final peptide concentration was measured at final concentration at A205 using NanoDrop One (ThermoFisher), and 150 ng was injected. The digest was separated using the NanoElute UHPLC system (Bruker Daltonics) with the Aurora Series Gen2 (CSI) analytical column (25 cm × 75 µm 1.6 µm FSC C18, with Gen2 nanoZero and CSI fitting; Ion Opticks, Parkville, Victoria, Australia) heated to 50°C and coupled to timsTOF Pro (Bruker Daltonics) operated in DIA‐PASEF mode. A standard 30‐min gradient was run, starting from 2% to 12% solvent B over the first 15 min, followed by an increase to 33% B from 15 to 30 min. The gradient was then ramped to 95% B over 0.5 min and held at 95% B for 7.72 min. Before each run, the analytical column was conditioned with 4 column volumes of buffer A. Where buffer A consisted of 0.1% aqueous formic acid and 0.5% acetonitrile in water, and buffer B consisted of 0.1% formic acid in 99.4% acetonitrile. The NanoElute thermostat temperature was set at 7°C. The analysis was performed at 0.3 µL/min flow rate. TimsTOF Pro was run with timsControl v. 4.1.12 (Bruker). LC and MS were controlled with HyStar 6.0 (6.2.1.13, Bruker).

#### 2.3.3 Search

Acquired library data were searched using FragPipe [50] computational platform with MSFragger [50, 51], Philosopher (v. 4.2.1 [52]), and EasyPQP (v. 0.1.27) components to build a spectral library. The protein sequence database for the honey bee (*A. mellifera*) from NCBI (2021) and common contaminant proteins, containing a total of 37,281 sequences, were used, where reversed protein sequences were appended to the original database as decoys. Precursor mass tolerance was set to 50 ppm and fragment mass tolerance to 20 ppm. Protease specificity was set to “trypsin,” with up to 2 missed cleavages. The MS/MS search results were further processed for each analysis using Philosopher, where final reports were generated and filtered at 1% protein FDR plus 1% PSM/ion/peptide‐level‐FDR [53]. Then the resulting reports were used as input to EasyPQP to generate consensus spectral libraries. The final library was filtered at a 1% FDR at the protein level.

For experimental samples, dia‐PASEF data were analyzed using parameters similar to those applied in the library search on FragPipe [50], with additional DIA quantification performed using DIA‐NN (v1.8 [54]). A spectral library generated from the previously mentioned highly fractionated samples was used for analysis. DIA‐quantification mode was set to “Any LC (high precision).” All other settings remained default. The mass spectrometry data were deposited to the ProteomeXchange via the MassIVE (Mass Spectrometry Interactive Virtual Environment) partner repository with the dataset identifier PXD062819.

#### 2.3.4 Pre‐Processing of Protein Intensity Matrix

We applied a two‐step procedure for feature selection. The data processing involved filtering rows from the original proteomics intensity table to ensure each sample contains at least two non‐missing values for either highbush blueberry or cranberry. The filtered data was then processed using log_2_ transformation to reduce skewness, followed by quantile normalization to standardize distributions across samples and minimize technical bias.

The protein intensity matrix has undergone the preprocessing steps; first the contaminant features, including common lab contaminants (e.g., keratin, trypsin, and actin) were removed from the dataset. Features present in at least 50% of the samples were retained for downstream analysis. For the remaining proteins, all missing values were imputed with a small number, except in cases where proteins were consistently identified in one ecosystem but not in another. In these instances, the mean values from the replicates were used as the imputed value for the expressed ecosystem. For the proteins that passed quality control and low‐abundance filtering, differential expression analysis was conducted using the limma package [55] in R (v 4.3.1) [56]. A linear model was applied to account for tissue type, year, and plate effects as covariates. Proteins with significantly different expression between the HBB and CRA ecosystems were identified using empirical Bayes moderation. Differentially expressed proteins (DEPs; HBB compared with CRA) were determined with an adjusted *p* value less than 0.05 (BH corrected [41]).

### 2.4 Microbiome

Gut sample processing, shotgun metagenomic sequencing, and taxonomic classification were performed as described in Tran et al. [57]. In total, 20 homogenized gut samples were sent to the Genome Quebec Centre of Expertise and Services (Montreal, Québec, Canada) for DNA extraction, library preparation, and paired‐end sequencing on a NovaSeq 6000 (Illumina). The 20 samples analyzed in this study had a mean read depth of 56.3 million paired‐end reads and a range of 40.8–84.0 million reads after quality control and the removal of PhiX bacteriophage reads.

For taxonomic classification, raw sequencing reads were adapter‐trimmed and quality‐filtered using Fastp (v0.23.2) [58]. Reads were taxonomically classified as bacterial using Kraken 2 (v2.1.2) [59] in conjunction with the BeeRoLaMa v1 Honey Bee Microbiota Database [57]. In total, 57 bacterial species were identified across all samples. On average, 12.3% of reads remained unclassified, with a range of 8.9% to 32.1%. In each sample, an average of 72.3% (maximum 79.2%) of reads were mapped to the host genome, while the remaining reads were assigned to bacterial taxa.

Analyses of microbial community composition, alpha‐ and beta‐diversity metrics, and statistics were performed using R. For microbiome analysis, only sequences from bacteria were used. Relative abundances were calculated, and genera with average proportions below 0.01% were excluded from downstream analysis. Rarefaction and diversity analysis of microbial community composition were performed with vegan [60] and base R packages. For principal coordinate analysis (PCoA), the data were normalized using centered log‐ratio (CLR) transformation via the “transform” function from the microbiome package [61, 62]. Euclidean distances were then computed using the “ordinate” function from the phyloseq package (v1.50.0) [63].

### 2.5 Pathogen and Agrochemical Analysis

From each colony, another batch of worker bees (*n* = 15 per colony) was sampled and pooled for pathogen [35] and agrochemical residue [15] analysis (Figure S1). Pathogen detection followed Office International des Epizooties (OIE) and Prevention of Honey Bee COlony LOSSes (COLOSS) Bee Book guidelines [64], with *Nosema* spores quantified via microscopy. While *Lotmaria passim* and major honey bee viruses (black queen cell virus = BQCV, deformed wing virus types A and B = DWV‐A and DWV‐B, Israeli acute paralysis virus = IAPV, and sacbrood virus = SBV) were assessed via real‐time qPCR, primers and detailed methods were available in a previous study in the Supporting Information of McAfee et al.’s paper [35]. Varroa mites were quantified using the alcohol wash method, and disease symptoms were recorded during colony inspections [36, 65]. Agrochemical residue analysis was performed using LC‐MS/MS at the Agriculture and Food Laboratory, University of Guelph, an ISO/IEC 17025 accredited laboratory. The analysis covered 232 unique pesticide compounds. Strict quality control measures, including method blanks, blind proficiency testing, and second‐person validation, were implemented to ensure data accuracy and prevent transcription errors.

### 2.6 Functional Annotation

Gene Ontology (GO) Term Enrichment Analysis and KEGG pathway enrichment analyses were performed using the differentially expressed proteins. The R package g:Profiler [66] was used to identify GO terms in biological processes (BP), molecular functions (MF), and cellular components (CC). Additionally, KEGG pathway analysis was conducted to explore functional pathways. GO terms and pathways with an FDR < 0.05 were considered statistically significant.

### 2.7 WGCNA Analysis

WGCNA was used to construct co‐expression networks and identify modules of co‐regulated proteins. A protein expression matrix was generated using normalized intensity data after regressing out the tissue, plates, and year effects, and the signed similarity matrix was calculated using Pearson correlation coefficients. Network construction was performed using the default parameters of WGCNA package in R [34]. Modules consisting of at least 30 proteins were retained for downstream analysis.

The first step of the co‐expression network construction involved calculating the eigengene based on the absolute expression values of the respective proteins. This approach was used to capture the dominant expression trend within each module, focusing on the overall direction of change across the blueberry versus cranberry comparison, regardless of whether individual proteins were upregulated in blueberry or cranberry. To better interpret modules with mixed expression patterns, submodules were generated. In some cases, proteins with opposing expression trends—i.e., proteins upregulated in the blueberry ecosystem and those upregulated in the cranberry ecosystem—were grouped within the same module due to their strong negative correlation patterns. This strong correlation suggests potential biological interactions, such as positive or negative feedback loops or compensatory mechanisms. To explore these interactions further, proteins that were upregulated in the HBB ecosystem and those upregulated in the CRA ecosystem were separated into distinct submodules. The eigengene for each submodule was then recalculated based on their expression values of the respective proteins. Subsequently, these recalculated submodule eigengenes were correlated with environmental and physiological traits, with their *p* values and correlation coefficients calculated.

Similarly, for transcriptomics analysis, proteins within each module were mapped to their corresponding genes, and RNA‐seq expression data were used to recalculate module eigengenes. Given that modules can contain genes with opposing expression trends, similar to the protein analysis, submodules were also generated for transcriptomic data. Genes that were upregulated in the HBB ecosystem and those upregulated in the CRA ecosystem were separated into distinct submodules. The eigengene for each submodule was recalculated to better represent the specific expression pattern of each group. The recalculated RNA‐derived submodule eigengenes were then correlated with traits, with their *p* values and correlation coefficients calculated to explore the associations between transcriptomic expression patterns and environmental or physiological factors.

### 2.8 Data Visualization

Geographic mapping was visualized using the plot_ordination function. A heatmap of the differentially expressed genes was generated using the heatmap.2 functions in the gplots [67] package and the pheatmap function in pheatmap [68] package. The heatmap of the co‐regulation of gene expression was generated using the TOMplot function in the WGCNA package in R. Bar charts for GO term and pathway enrichment analysis were generated using GraphPad Prism v 8.0.2 [69]. Module‐trait correlation plots were made using functions in WGCNA packages, showing only significant correlations. Plots in microbiome analysis were produced using ggplot2 [70].

## 3 Results

### 3.1 Measuring Pollination Ecosystem Effects on Honey Bee Gene Expression

To explore global gene expression profiles across various bee tissues under blueberry and cranberry pollination ecosystems, we conducted an integrative analysis of RNA and protein data. Among the transcriptomic data, we identified transcripts assigned to 11,334 genes, of which 9258 were protein‐coding. Proteomic profiling identified 5893 unique proteins, mapped to 5687 genes. Both transcriptome and proteome data were highly reproducible, with Pearson correlation scores typically greater than 0.9 (Figures S2 and S3a).

### 3.2 The Proteome and Transcriptome Atlases of Bees Exposed to Blueberry and Cranberry Ecosystems

**FIGURE 2:**
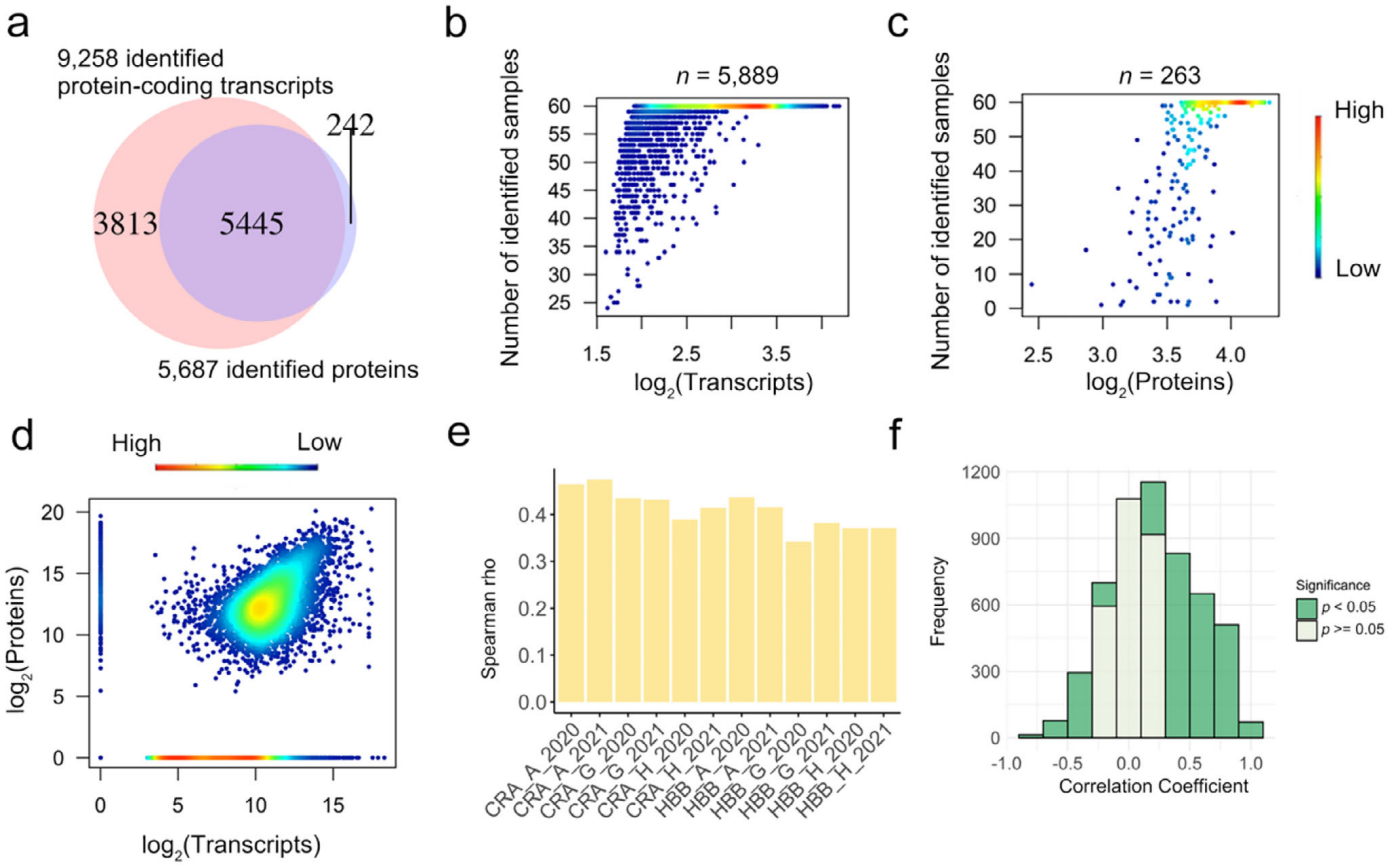
The proteomic and transcriptomic atlases of bees exposed to blueberry and cranberry ecosystems from three tissues across 2 years. (a) Venn diagram of the identified gene numbers at the protein (purple) and mRNA (red) levels among the two ecosystems. (b,c) Density distribution for the transcripts of missing proteins (b) and the protein products of missing mRNA transcripts (c) according to their average expression values and identification frequencies across the two ecosystems. The color scale (blue to red) represents density from low to high, with warmer colors (yellow/red) indicating more frequent transcripts/proteins detection at higher expression levels. (d) The overlapping genes between mRNA and proteome, with a density scatterplot illustrating protein intensities versus mRNA abundance, based on the mean copy number values. The color scale (blue to red) represents density from low to high. (e) Histogram showing the Spearman correlation coefficients between the average expression levels of matched proteins and transcripts for each group. There are 20 groups spanning two ecosystems (CRA and HBB), three tissues (abdomen [A], gut [G], and head [H]), and two sampling years (2020 and 2021). Each bar represents one group's transcriptome–proteome correlation, calculated using five biological replicates per omics layer. Correlation values were computed using a two‐sided Spearman's rank test, based on genes jointly detected in both datasets. (f) Histogram showing the distribution of gene‐wise Spearman correlation coefficients between RNA and protein expression levels (two‐sided Spearman's rank correlation test, *p* value < 0.05), highlighting genes with high or low RNA‐to‐protein correlation.

Joint analysis of transcriptomic and proteomic datasets revealed substantial overlap in gene product detection. A total of 5445 out of 9258 protein‐coding genes whose transcripts were detected were also detected at the proteome level (Figure 2a). Notably, the identification of 5445 proteins constitutes one of the most comprehensive proteomic datasets generated to date for *A. mellifera*. The transcriptome had a wider spread, with more frequent lower values, while the proteome had a tighter distribution around a slightly higher value range, which aligns with biological expectations—transcripts are typically more variable, while protein profiling is more stable and regulated (Figure S3b). Due to the wider range of expression levels at the protein level versus the transcript level and the fundamental differences in how proteins and transcripts are detected, the ratio of proteins:transcripts is roughly what would be expected based on prior studies, which show that proteomics typically captures 30%–50% of corresponding transcripts due to differences in stability and detection sensitivity [71, 72].

To systematically determine the differences between the detectable proteome and the transcriptome, we compared the expression levels of co‐identified and exclusively identified genes between two omics datasets (Figure 2b,c). Transcripts from 5889 genes were identified without corresponding protein detection, including a large number of metabolic and motor proteins that were not expected to be functionally expressed in these bee populations (Figure S3c,d). While one would expect to identify some transcripts where the encoded protein was not detected, it is less typical to identify a protein without finding the transcript. Even so, we identified 242 genes whose corresponding proteins (*n* = 263) were detected, but no matching transcripts were found. These proteins tended to be expressed at moderate levels, although they were not universally detected across all samples. This discrepancy may arise from multiple factors, including differences in molecular half‐lives, detection sensitivity, translational control, and sample heterogeneity. Notably, long‐lived proteins can persist after their mRNAs have degraded, while low‐abundance transcripts may fall below RNA‐seq detection thresholds. And most of these proteins were related to monoatomic ion transmembrane transport as well as the immune‐related Toll and Imd signaling pathways (Figure S3c,d), which are essential for innate immune responses in honey bees. These pathways might particularly rely on rapid, post‐transcriptionally regulated responses, allowing bees to quickly adapt to pathogen exposures and environmental stresses. Further targeted studies involving sRNA sequencing, protein stability assays, mRNA degradation rates, or ribosome profiling could help clarify these regulatory mechanisms.

We then surveyed both the gene‐wise and sample‐wise correlations of 5445 co‐identified genes in the transcriptome and proteome. The results revealed moderate correlations between quantified transcripts and proteins among different groups of samples (Spearman correlation coefficient rho values from 0.35 to 0.45) (Figure 2e). Only 24% of the proteins displayed a significant correlation with the cognate RNA (1322 proteins, Spearman rho > 0.5 or Spearman rho < −0.5, *p* value < 0.05) (Figure 2f).

### 3.3 Dysregulation of Proteins and Pathways in the Bees Exposed to Two Crop Ecosystems

**FIGURE 3:**
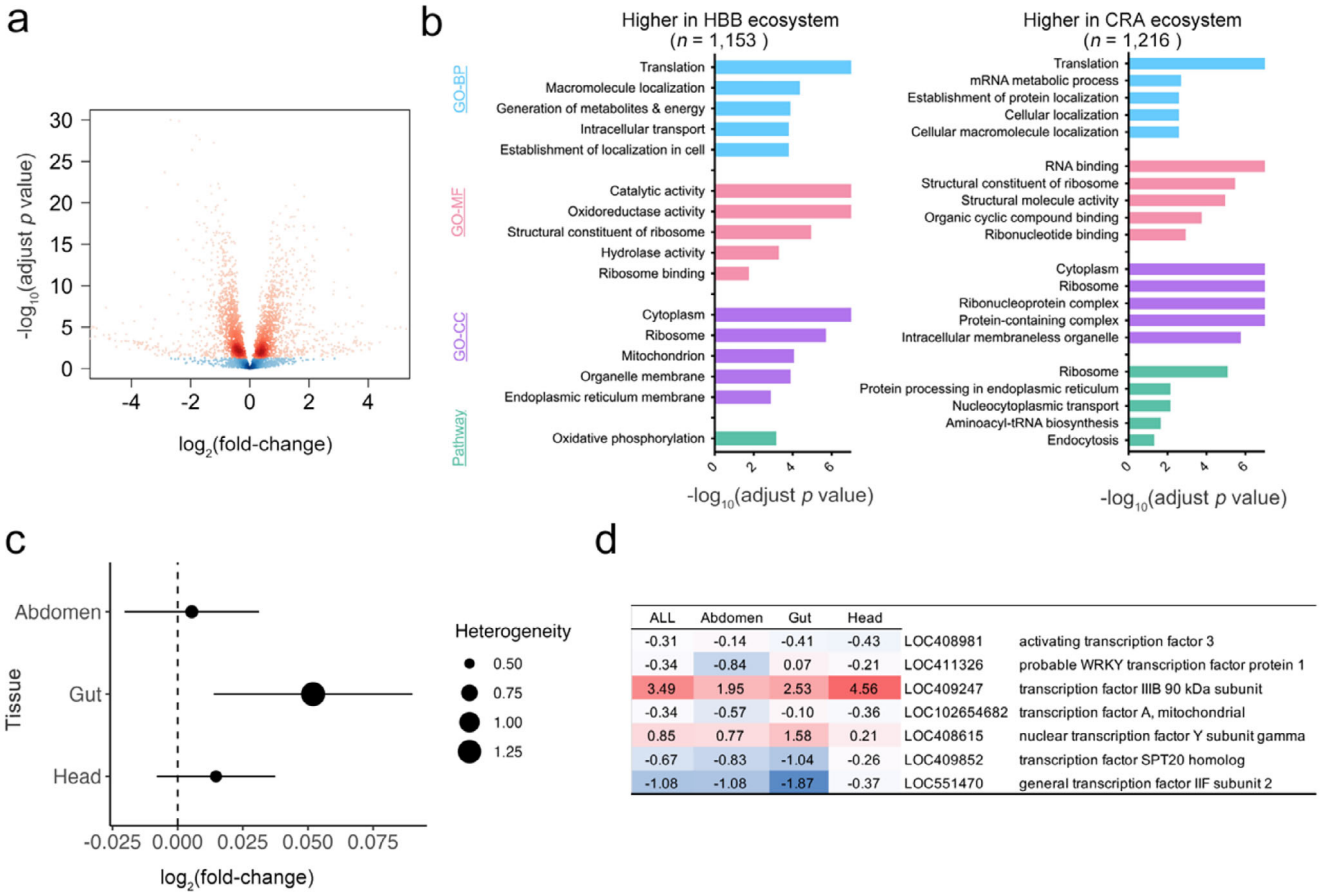
Proteins and pathways that were alternatively regulated in blueberry ecosystems compared with cranberry ecosystems. (a) Volcano plot depicting significantly differentially expressed proteins (sigDEPs) in blueberry ecosystem compared with cranberry ecosystem, adjusted for tissue and years. Red color indicates the significantly differentially expressed proteins with adjusted *p* value less than 0.05. (b) GO term enrichment analysis for biological process, molecular function, and cellular component, along with pathway enrichment analysis for proteins with higher abundance in the HBB ecosystem and proteins with higher abundance in the CRA ecosystem. (c) Meta‐analysis of differential expression between ecosystems across three tissue types. The *τ* ^2^ was denoted as heterogeneity, and the log_2_ fold change (effect size), as well as the 95% confidence intervals, were shown. (d) Transcription factors found in the sigDEPs, with their log_2_ fold change in all tissues or within specific tissues displayed.

To understand the molecular changes in bee physiology when acting as pollinators of different crop systems, we began by examining the differences at the proteome level. After preprocessing mentioned in the Methods section, we analyzed 4101 proteins and identified dysregulations between the two bee populations using a linear model that incorporated tissue, plate effects, and year as covariates. Not surprising given the differences in both crop and time, 2369 significantly differentially expressed proteins (sigDEPs) were identified in the blueberry ecosystem compared with the cranberry ecosystem, including 1153 proteins with higher abundance in the blueberry ecosystem and 1216 proteins with higher abundance in the cranberry ecosystem (adjusted *p* value < 0.05; Figure 3a). We subsequently conducted GO term enrichment analysis to examine the GO terms and pathways associated with those differentially expressed proteins. The proteins upregulated in the blueberry ecosystem were involved in the biological processes including translation (FDR = 3.31 × 10^−9^), localization (FDR = 2.84 × 10^−5^ to 1.58 × 10^−4^), generation of precursor metabolites and energy (FDR = 1.31 × 10^−4^), intracellular transport (FDR = 1.58 ×10^−4^), and oxidative phosphorylation pathways (FDR = 6.91 ×10^−4^) (Figure 3b). And the proteins upregulated in the cranberry ecosystem were involved in the translation (FDR = 7.27 × 10^−7^), mRNA metabolic process (FDR = 1.99 × 10^−4^), localization (FDR = 2.60 × 10^−3^) and other biological processes, as well as in the pathways such as ribosome (FDR = 8.12 ×10^−6^), Protein processing in endoplasmic reticulum (FDR = 7.09 × 10^−3^), nucleocytoplasmic transport (FDR = 7.09 × 10^−3^), aminoacyl‐tRNA biosynthesis (FDR = 2.25 × 10^−2^), and endocytosis (FDR = 4.81 × 10^−2^) pathways (Figure 3b). Proteins that were upregulated in either the blueberry or cranberry ecosystem were independently enriched for the biological process “translation.” This dual enrichment likely reflects the complex, multi‐component nature of the translational machinery, which involves numerous genes operating in distinct sub‐processes (e.g., initiation, elongation, termination, and ribosome assembly). Different subsets of these components may respond in opposite directions under varying environmental stressors and energetic demands, leading to their concurrent enrichment in both the blueberry‐upregulated and cranberry‐upregulated groups. Together, these findings suggest that bees foraging in blueberry ecosystems exhibit enhanced metabolic and transport activity, potentially reflecting increased energetic demand, whereas those in cranberry ecosystems show stronger enrichment for biosynthetic and translational machinery, possibly indicating cellular stress responses or recovery processes. Hence, by identifying which proteins and pathways are altered in two populations of bees, these results reflect the presence of molecular responses in honey bees associated with their environmental context.

PCA plot shows that there was more difference between tissues for the same ecosystem than between ecosystems of the same tissue (Figure S4). Through meta‐analysis, the overall heterogeneity varies across tissues (head = 0.45, gut = 1.3, abdomen = 0.58) (Figure 3c, Figure S4), indicating that the crop systems’ differences are relatively inconsistent across the different tissue types. Gut samples exhibited a larger τ^2^ value (denoted as heterogeneity score), indicating greater variability in the effect size compared to the head and abdomen tissues. This suggests that the effect observed in the gut is less consistent across studies or conditions, suggesting greater variability and potential context‐dependence. We also observed seven transcription factors differentially expressed in the proteome, with their changes within the ecosystems (HBB/CRA) consistent across three tissues (Figure 3d).

Notably, our transcriptomic profiling uncovered ecosystem‐specific changes (HBB/CRA) not only in protein‐coding transcripts but also in small regulatory RNAs (sRNAs), including long non‐coding RNAs (lncRNAs) and small nuclear RNAs (snRNAs), suggesting their potential roles in environmental adaptation. Approximately 7% of the significantly differentially expressed transcripts were annotated as lncRNAs, while small fractions (<1%) included non‐translating coding sequences, ribosomal RNAs (rRNAs), and snRNAs (Figure S5). Although our analysis mainly focused on protein‐coding genes, these non‐coding RNAs (ncRNAs) may exert important regulatory functions. lncRNAs can modulate gene expression at multiple levels, and snRNAs are involved in splicing, potentially influencing transcript diversity under agroecosystem‐related stress.

### 3.4 Comparative Proteome Analysis of Bee Colonies Revealed the Tissue‐Specific and Tissue Non‐Specific Functions in Different Ecosystems

**FIGURE 4:**
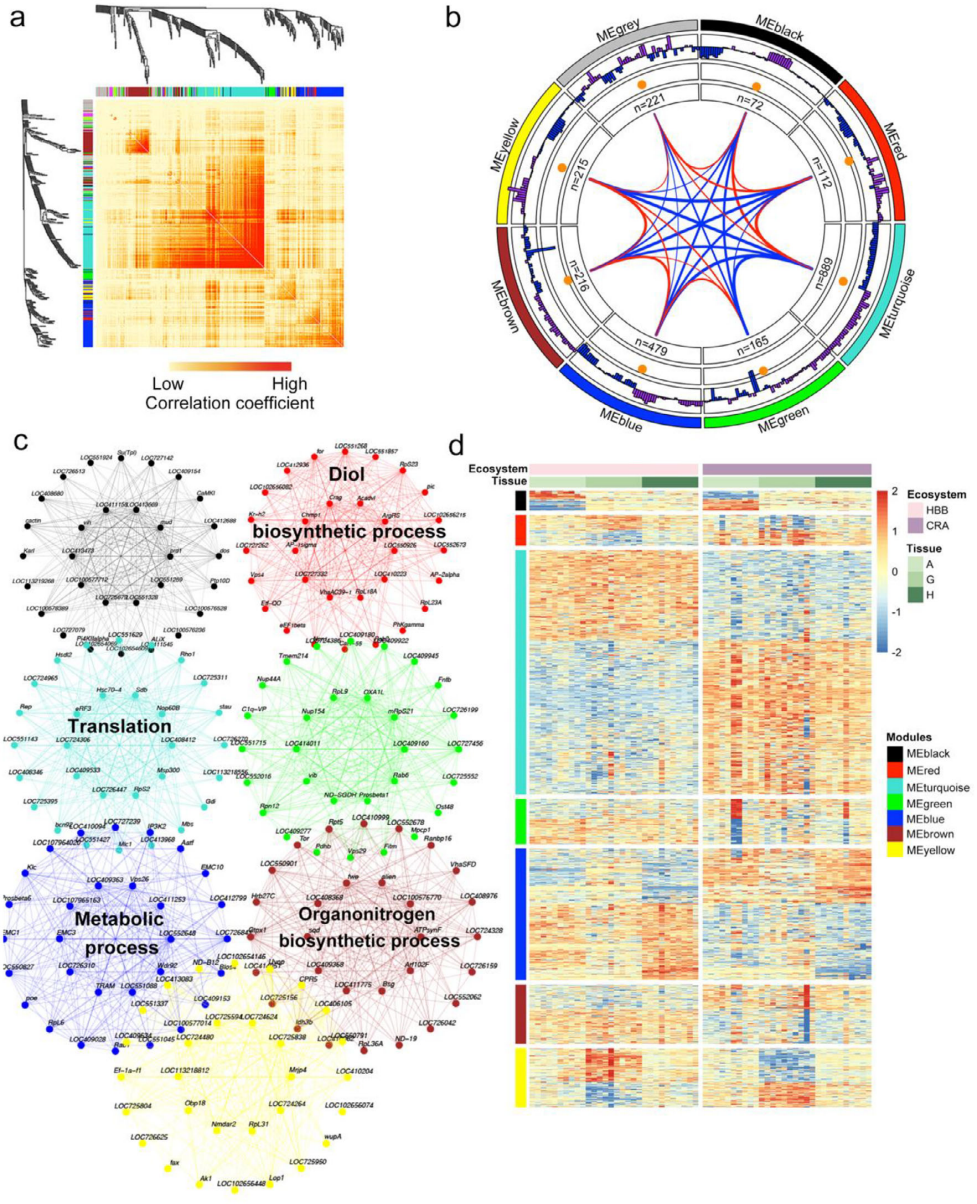
Classification of identified modules as tissue‐specific or non‐specific and their associated pathways. (a) Topological overlap matrix (TOM) plot showing the hierarchical clustering of 2369 differentially expressed proteins across all samples. The heatmap illustrates pairwise correlation coefficients among proteins, with module colors aligned on the top and left axes representing identified protein co‐expression modules. (b) Circos plot visualizing four concentric data layers of WGCNA protein modules: Outer ring: Colored sectors represent individual WGCNA modules; each color corresponds to a module. Module names are displayed within each sector. Second ring: Bar plots show the eigengene values (i.e., first principal component of protein expression) for each sample within the module. Bars above the axis (purple) indicate positive eigengene values, while bars below the axis (blue) indicate negative values. Samples are ordered by group: HBB‐Abdomen, HBB‐Gut, HBB‐Head, CRA‐Abdomen, CRA‐Gut, CRA‐Head. Third ring: Orange dots represent the average eigengene value for each module across all samples. Fourth ring: Text annotations indicate the number of proteins (n) assigned to each module. Links between modules: Lines connecting modules represent the pairwise correlations between module eigengenes. Line thickness encodes the strength of the correlation. Red lines denote positive correlations, and blue lines indicate negative correlations. (c) Representative biological processes enriched in each module, based on Gene Ontology term enrichment analysis. Nodes represent individual proteins within a given module, with edges denoting co‐expression relationships. (d) Heatmap of normalized protein expression profiles within each module across ecosystems (HBB, CRA) and tissue types (head, gut, abdomen). *Z*‐score scaling was applied per protein. Rows are grouped by protein expression within each module and color‐coded on the left; columns are annotated by ecosystem and tissue type above.

Given the involvement of the above‐mentioned differentially expressed proteins in diverse biological pathways, it is important to identify protein modules to focus on understanding the key biological processes for bees in different ecosystems. As genes involved in a common biological process often share regulatory mechanisms and expression patterns, we applied WGCNA to identify co‐regulated protein modules. These modules exhibited altered expression between blueberry and cranberry ecosystems and showed high intra‐module correlation. In brief, we constructed a co‐expression network (Figure 4a) from the 2369 differentially regulated proteins in the bee samples and identified seven protein modules, which we designated MEblack, MEred, MEturquoise, MEgreen, MEblue, MEbrown, and MEyellow (Figure 4b). Module sizes ranged from 72 to 889 proteins. For each module, the first principal component—referred to as the “eigengene”—was calculated and visualized as bar graphs in the Circos plot (Figure 4b).

Gene Ontology analysis revealed that many of these gene modules are associated with biosynthesis and metabolic pathways (Figure 4c). For example, MEturquoise is in Ribosomal Structure & Protein Synthesis (FDR = 1.12×10^−12^), MEbrown is involved in Protein Transport & Degradation (FDR = 3.56 ×10^−2^), and MEred in Diol biosynthetic process (FDR = 1.77 × 10^−2^), MEblue in Organophosphate metabolic process (FDR = 2.62 ×10^−2^), and MEyellow in Oxidoreductase activity (FDR = 2.15 ×10^−2^). Hub genes (biomarkers) were selected using module membership *p* values (MMp) less than 0.05 and module membership (MM) scores larger than 0.8, denoted as the potential biomarkers in each module (Figure 4c). These results highlight that different ecosystems not only drive tissue‐specific molecular responses in honey bees but also alter core functional networks related to protein synthesis and energy metabolism. The identification of ecosystem‐associated protein modules and their hub genes provides candidate molecular biomarkers for understanding how crop environments shape bee physiology.

Modules were then classified as either tissue‐specific or tissue non‐specific (Figure 4d). The MEblack module exhibited considerable variance between ecosystems in the abdomen. Besides, the MEred module displayed the highest variance in the gut. The MEblue module showed the greatest variance between ecosystems in both the abdomen and head. In contrast, the MEturquoise and MEbrown modules did not demonstrate strong tissue specificity. Overall, our network‐based analysis reveals how ecosystem context modulates core proteomic programs in honey bees, providing insight into the biological processes driving resilience or vulnerability across tissues.

### 3.5 Associations Between Pesticides and Pathogens in Bee Protein Profiles (Figure 5)

**FIGURE 5:**
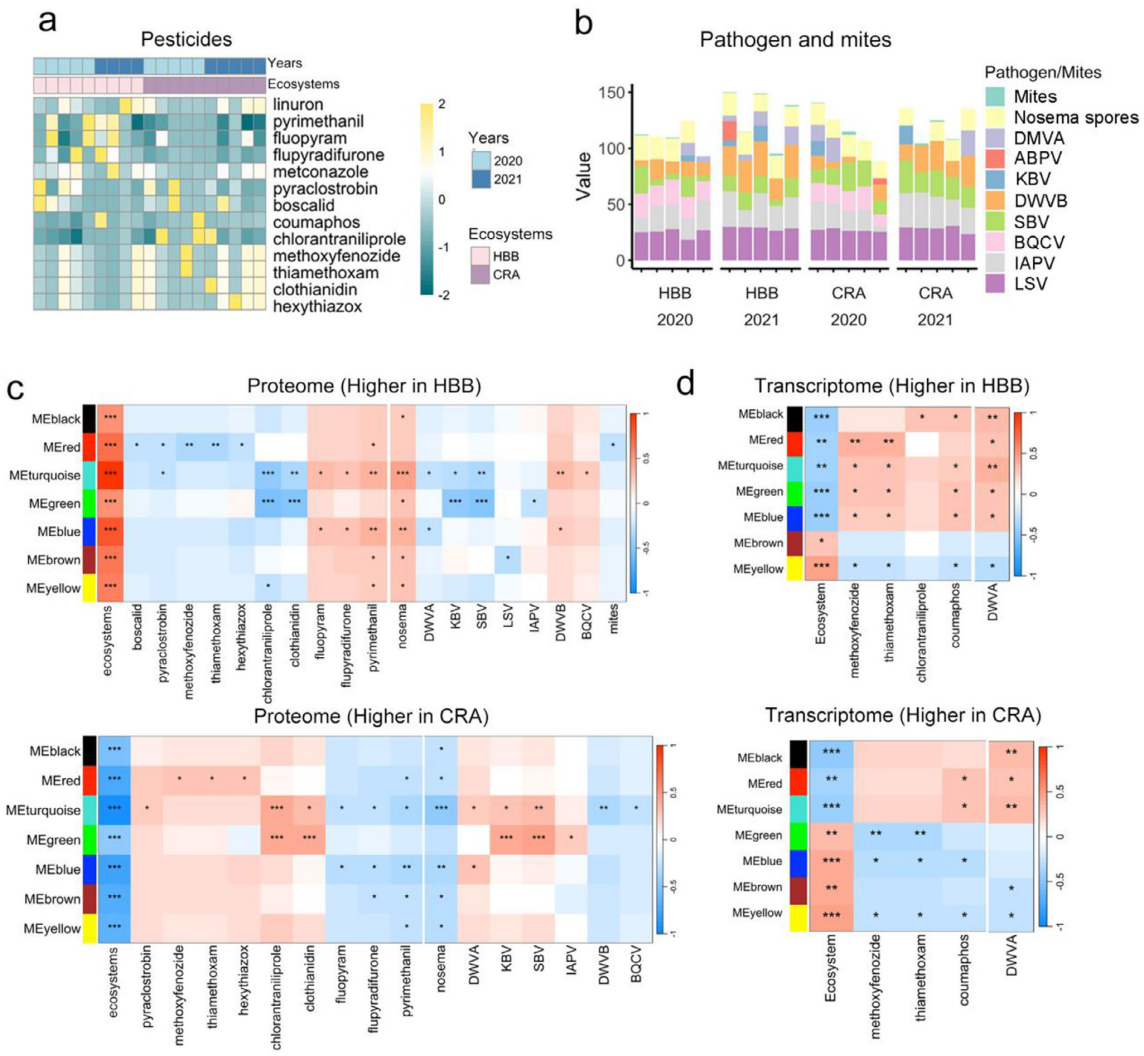
Effects of pesticide and pathogen/mite exposure on bee protein and transcript profiles. (a) Pesticides: Heatmap representing normalized relative pesticide residues detected in pooled bees from each colony across 2 years (2020, 2021) and ecosystems (HBB, CRA). Residues are grouped by pesticide class: herbicides (e.g., linuron), fungicides (e.g., pyrimethanil, fluopyram, metconazole, pyraclostrobin, and boscalid), miticides (e.g., coumaphos, chlorantraniliprole, and hexythiazox), and insecticides (e.g., clothianidin, flupyradifurone, thiamethoxam, and methoxyfenozide). Color intensity indicates z‐score transformed values; negative values represent below‐average concentrations relative to other samples. (b) Pathogen and mites: Stacked bar chart showing the relative abundance of major honey bee pathogens and Varroa mites across replicates, grouped by ecosystem (HBB and CRA) and year (2020, 2021). Each color represents a distinct pathogen or parasite (e.g., *Nosema* spp., *Varroa destructor*, DWV, LSV, etc.). The *Y*‐axis shows standardized abundance values (*Z*‐scores) derived from original pathogen quantification datasets (e.g., mite counts, spore counts, and viral load from qPCR Ct values). This normalization ensures comparability across taxa with different units. (c) Proteomics: Heatmap showing module–phenotype correlations derived from WGCNA. Rows represent protein modules identified from the proteome data, and columns represent phenotypic traits (e.g., pesticides, pathogens and so on). The color scale indicates correlation strength (red for positive and blue for negative correlations), with significance levels denoted by asterisks (*p value < 0.05, **p value < 0.01, ***p value < 0.001). For each protein co‐expression module, proteins were stratified based on direction of change (upregulated in CRA or upregulated in HBB ecosystems). Eigengene values were separately calculated for each subgroup and correlated with environmental and physiological traits. The upper panels show modules with higher protein expression in the CRA ecosystem; the lower panels show those elevated in the HBB ecosystem. (d) Each transcriptomic module was constructed using the same gene sets as the corresponding proteomic modules shown in panel c, based on matched gene identifiers. Module eigengenes, representing the first principal component of expression within each gene module, were correlated with phenotypic traits including ecosystem type, pesticide exposure, and pathogen levels. The color scale and significance annotations follow the same conventions as in panel c, highlighting concordance between proteomics and transcriptomics.

The interplay between pesticides and pathogens may be critical in influencing bee health, potentially serving as both cause and effect in the observed physiology dynamics. In our pesticide screening (Figure 5a), herbicides, fungicides, miticides, and insecticides displayed ecosystem‐specific and year‐specific variation in abundance, reflecting differences in local crop management practices and chemical application regimes. We observed relative shifts in pathogen profiles between ecosystems, including lower standardized *Varroa* mite abundance but higher *Nosema* levels in the blueberry compared to cranberry ecosystems (Figure 5b).

#### 3.5.1 Protein Co‐Expression Networks

To further dissect ecosystem‐specific patterns, we stratified the proteins in each co‐expression module into two subgroups based on their direction of differential expression: those upregulated in the CRA ecosystem and those upregulated in the HBB ecosystem. For each subgroup, we recalculated eigengene values—representing the first principal component of protein expression within that subgroup—and assessed their correlations with key environmental and physiological traits (e.g., pesticide residues, pathogen load, ecosystem identity) (Figure 5c). To avoid dominant tissue‐specific effects masking environmental signals, tissue identity (along with plate and year) was regressed out prior to this analysis. This ensured that the observed correlations more directly reflected ecosystem‐driven molecular responses.

Further analysis identified specific modules that correlated with higher levels of pesticides, suggesting possible synergistic effects that impact bee activity. For example, the pesticides pyrimethanil, fluopyram, and flupyradifurone showed positive correlations with the MEturquoise module, which was higher expressed in HBB. In contrast, chlorantraniliprole and clothianidin were negatively correlated with the same module. Conversely, the MEturquoise module with higher expression in CRA exhibited the opposite patterns of association.

In addition, pathogens, including mites and some bee viruses, also had disparate relationships with the identified protein modules (Figure 5c). For instance, the MEturquoise module (higher in HBB) was positively correlated with *Nosema* and two viruses (DWV‐B, BQCV), but negatively associated with three other viruses (DWV‐A, KBV, SBV). The MEred module was negatively associated with mite infestations but positively associated with *Nosema*.

Of particular interest, the MEgreen module demonstrated a strong negative correlation with two pesticides (chlorantraniliprole and clothianidin) and two viruses (KBV and SBV), highlighting potential protective or mitigating factors within this protein module.

#### 3.5.2 Protein and RNA Co‐Expression Networks

Correlation analyses using module eigengenes from co‐expression networks revealed significant but opposing trends between proteomics and transcriptomics data in response to pesticide and pathogen exposure (Figure 5d).

In the MEturquoise module, a significant correlation with DWV‐A was observed in both datasets. While protein expression levels in the HBB or CRA ecosystems showed a significant increase, the corresponding transcriptomic data demonstrated a significant negative correlation. This inverse relationship suggests potential post‐transcriptional regulation or compensatory mechanisms in response to viral infection. In the MEred module, significant positive correlations were identified for the pesticides methoxyfenozide and thiamethoxam in both the proteome and transcriptome data. However, the trends were reversed: negative correlations between these two pesticides and protein levels were detected in the HBB ecosystem, while transcript levels exhibited significant positive correlations. This divergence indicates possible regulatory feedback mechanisms or protein‐level stabilization despite transcriptional suppression.

These findings suggest that honey bee molecular responses to environmental stressors involve complex regulatory processes. The significant but opposing trends between transcriptomic and proteomic responses highlight the need for integrative analyses to capture the full scope of ecosystem‐specific adaptations. Further functional investigation into the pathways within the MEturquoise and MEred modules may provide insights into the molecular mechanisms underlying pesticide and pathogen interactions.

### 3.6 Specific Microbiomes Were Correlated with Protein Modules (Figure 6)

**FIGURE 6:**
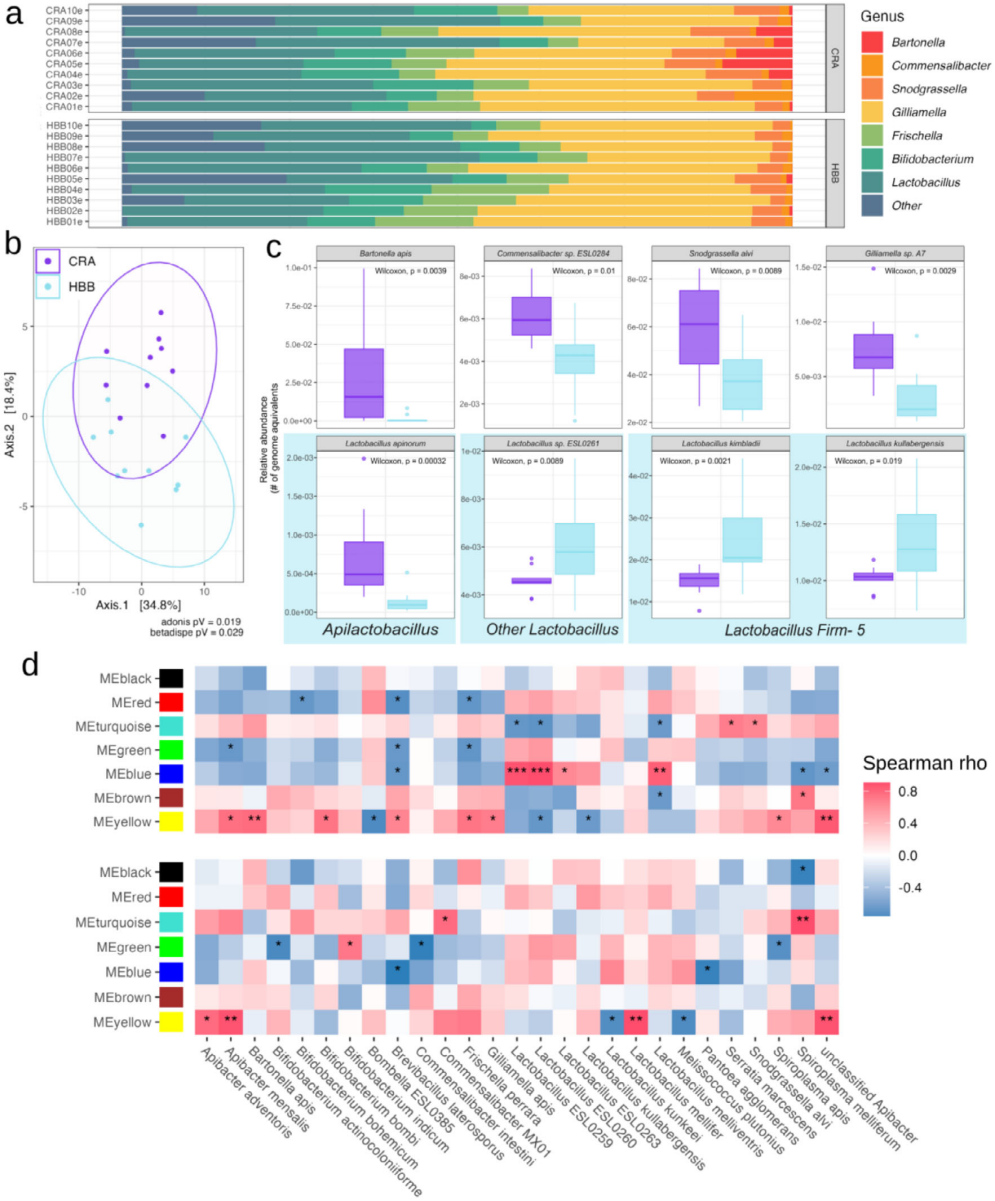
Relationship between proteome and microbiome. (a) Barplots with relative bacterial abundance in the guts of bees from HBB or CRA ecosystems on the genus level. (b) PCoA based on Aitchison distance dissimilarities on the gut communities of different ecosystems based on Kraken 2‐classified shotgun metagenomes. (c) Box‐plots with relative bacterial abundance in the guts of bees from the two ecosystems expressed as the number of genome equivalents calculated with Kraken 2. Only taxa with significantly different levels were plotted (for not‐significant bacteria see Supporting Information) on the species level. (d) Spearman rho heatmap showing associations between microbial taxa abundance and proteomic co‐expression modules identified by previous WGCNA analysis. Only taxa with significant correlations are annotated with asterisks (*p value < 0.05, **p value < 0.01, ***p value < 0.001). Each ME module is color‐coded and represents a group of co‐expressed proteins. The upper panel shows proteomic modules that are up‐regulated in the HBB ecosystem, while the lower panel represents those up‐regulated in the CRA ecosystem.

Microbiome classification using the BeeRoLaMa v1 database identified core bacterial genera in the honey bee gut, including *Bifidobacterium*, *Gilliamella*, *Snodgrassella*, *Lactobacillus* Firm5, and *Lactobacillus* Firm4 (Figure 6a). Additionally, non‐core genera such as *Commensalibacter*, *Frischella*, and *Bartonella* were detected [73, 74, 75]. The relative abundance of these core genera was consistent with previously reported bee microbiomes, where *Gilliamella* and *Snodgrassella* dominated, particularly in bees during May–July [76]. Lower levels of *Lactobacillus* and *Bifidobacterium* further supported these expected microbial distributions.

A permutation multivariate analysis of variance revealed significant differences in gut microbial composition between bees from HBB and CRA agroecosystems (adjusted *p* value = 0.019, *r* ^2^ = 0.13). PCoA (Figure 6b) demonstrated distinct clustering of microbial communities, confirming that gut microbiomes differ significantly between the two crop environments. Notably, microbiome compositions did not show significant interannual variation (*p* value > 0.05, Figure S6), suggesting that these microbial differences are primarily driven by crop‐specific environmental factors rather than year‐to‐year fluctuations.

Differential abundance analysis (Figure 6c) identified microbial taxa with significant shifts in bees from blueberry and cranberry ecosystems (adjusted t‐test *p* value < 0.05). Key differentially abundant species included *Bartonella apis*, *Commensalibacter* spp. ESL0284, *Snodgrassella alvi*, *Gilliamella apicola*, and several species from the *Lactobacillus* genus.

To further explore functional interactions, we examined correlations between microbial taxa and proteomic modules. Heatmap analysis (Figure 6d) revealed significant associations between gut microbiota and proteomic profiles, highlighting potential microbiome‐driven effects on honey bee metabolism and immune function.

Additionally, microbial associations with pesticides and pathogens (Figure S7) further support the hypothesis that gut microbiota influence honey bee resilience to environmental stressors. Certain bacterial species displayed strong correlations with pesticide residues and pathogen loads, suggesting potential microbiome‐mediated processes or pathogen susceptibility, which also were shown in other studies [66, 67, 68, 70, 77, 78]. These findings demonstrate that honey bee gut microbiomes vary significantly between ecosystems and are functionally linked to host proteomic responses.

## 4 Discussion

Our study presents a novel multi‐omics framework for investigating honey bee health, integrating proteomics, transcriptomics, and microbiome profiling across distinct tissues to capture the complex interplay of stressors in agricultural environments. By employing a module‐based, systems biology approach, we connect multi‐omics datasets to identify key regulatory networks and stress‐response pathways, providing a deeper understanding of environmental determinants of bee resilience. This integrative strategy enables a more precise characterization of stressor interactions that would be overlooked in traditional whole‐body studies, highlighting the potential for omics‐based biomarkers in pollinator conservation.

Honey bee populations exhibit a high degree of genomic similarity across global regions. Although some geographic variation exists, overall genomic differences among *A. mellifera* populations are relatively minor [79, 80]. Therefore, in bees originating from different regions but exposed to similar agroecosystem contexts, gene expression patterns are more likely to reflect ecosystem‐level factors (e.g., crop type, pesticide exposure, nutritional resources) rather than regional genetic background.

Although our study includes colonies placed in different crop systems (e.g., highbush blueberry vs. cranberry), we do not interpret the observed differences in bee physiology as a direct effect of crop species per se. Instead, we interpret the observed omics signatures as emergent outcomes of ecosystem‐level exposures, which encompass not only the crop type but also the surrounding landscape, management practices (e.g., pesticide use), and pathogen pressures. Indeed, previous studies [81] have shown that pesticide risk during blueberry pollination can be driven by off‐farm exposures. Thus, the differences between highbush blueberry and cranberry ecosystems may reflect the integrated agroecological context in which bees operated, rather than intrinsic properties of the crop alone.

A key innovation in our study is the use of co‐expression network analysis to integrate proteomic, transcriptomic, and microbiomic data into shared functional modules. Rather than analyzing each omics layer in isolation, we applied WGCNA to identify modules of co‐regulated features across tissues and modalities, allowing us to uncover coordinated molecular responses to environmental stressors. This network‐based approach enabled cross‐modal integration and revealed functionally connected patterns linking host gene and protein expression with microbial composition.

Several modules identified through co‐expression networks showed consistent associations with immune‐related stressors, including key honey bee viruses (e.g., DWV, SBV) and immunomodulatory pesticides (e.g., thiamethoxam). Notably, the MEred and MEblack modules exhibited strong correlations with both pathogen load and pesticide residues in both the proteome and transcriptome layers (Figure 5c,d), suggesting that these co‐regulated gene/protein clusters may represent a potential core role in immune response.

Our study provides new molecular evidence supporting the hypothesis that pesticide exposure exacerbates pathogen susceptibility in honey bees. We detected multiple agrochemicals—including neonicotinoids, fungicides, and miticides—in bee tissues, with significant correlations between pesticide presence and proteomic alterations in metabolic pathways. Notably, increased *Nosema* prevalence in pesticide‐exposed bees suggests that agrochemicals may compromise immune defenses, facilitating pathogen proliferation [31].

While our study provides novel insights into honey bee health, several limitations must be acknowledged. First, our data were collected over a 2‐year period; extended longitudinal studies will be essential to assess interannual variability and the lasting effects of environmental stressors. Second, although our multi‐omics approach captures broad molecular changes, additional functional validation of differentially expressed genes and proteins is necessary to establish causal relationships.

The microbiome analysis further underscores these differences. We observed a higher abundance of *S. alvi* and *G. apicola* in bees from cranberry fields, which are bacterial taxa that have been involved in metabolic pathways and pathogen protection [23]. This shift in microbial composition aligns with previous research [23, 82] and might indicate that gut microbiota play a crucial role in host adaptation to environmental stressors (Figure S7), including agrochemical exposure and dietary variation.

Future research should investigate the effects of specific agrochemical formulations on honey bee physiology across different developmental stages and seasonal conditions. Given that nutritional availability fluctuates with floral phenology, agrochemicals may exert both acute and cumulative effects, and pathogen prevalence can vary seasonally, it is essential to consider time as a dynamic component of environmental exposure. Longitudinal, multi‐season studies are therefore needed to assess the delayed and additive impacts of agroecosystem conditions. However, tracking colonies across extended periods poses substantial complexity, as bees continually transition through changing floral sources and environmental contexts, potentially confounding causal inference. Expanding the microbiome analysis to include functional metagenomics could provide deeper insights into the role of gut bacteria in detoxification and immune modulation. Furthermore, comparative studies involving wild pollinators would help determine whether similar molecular adaptations occur across different bee species, contributing to broader pollinator conservation efforts.

In conclusion, our study presents a comprehensive system‐level analysis of honey bee health in blueberry and cranberry agroecosystems, integrating proteomics, transcriptomics, and microbiome profiling through a module‐based network approach to elucidate the complex interactions between environmental stressors and pollinator physiology. By uncovering tissue‐specific molecular responses and identifying key regulatory pathways, our findings advance the understanding of honey bee resilience and inform science‐based conservation strategies. Moving forward, the integration of multi‐omics data into pollinator health assessments will be crucial for developing targeted interventions to mitigate environmental stressors and ensure the sustainability of agricultural pollination services.

## Author Contributions

Conceptualization: Huan Zhong, Leonard J. Foster, Amro Zayed, M. Marta Guarna, Stephen F. Pernal, RC, Pierre Giovenazzo, Shelley E. Hoover. Methodology: Huan Zhong, Yuming Shi, Jason C Rogalski, Amanda S. Gregoris, Heather Higo, Julia Common, Ida M. Conflitti, Renata Moravcova, Mateus Pepinelli, Lan Tran, Amro Zayed, Leonard J. Foster, and M. Marta Guarna. Validation: Huan Zhong, Yuming Shi, and Amro Zayed. Formal analysis: Huan Zhong, Yuming Shi, Aleksandra Kozlova, Jason C Rogalski, Lance Lansing, Renata Moravcova, Kyung‐Mee Moon, Xiaojing Yuan and Aidan Jamieson. Data curation: Huan Zhong, Ida M. Conflitti, Mateus Pepinelli, Syed Abbas Bukhari, Sarah K. French, Lan Tran, Renata Moravcova, Heather Higo, Julia Common, and Jason C. Rogalski. Writing – original draft: Huan Zhong, Yuming Shi, Aleksandra Kozlova, Leonard J. Foster, Renata Moravcova, and Jason C. Rogalski. Writing – review and editing: all. Funding acquisition: Leonard J. Foster, Amro Zayed, Shelley E. Hoover, Stephen F. Pernal, Pierre Giovenazzo, and M. Marta Guarna. Resources: Amro Zayed, Pierre Giovenazzo, Leonard J. Foster, M. Marta Guarna, and Huan Zhong. Project administration: Amro Zayed and Leonard J. Foster. Supervision: Leonard J. Foster, Amro Zayed, M. Marta Guarna, Stephen F. Pernal, Shelley E. Hoover, Pierre Giovenazzo, and RC.

## Conflicts of Interest

The authors declare no conflicts of interest.

## Supporting information




**Supporting File**: pmic70033‐sup‐0001‐SuppMat.docx.

## Data Availability

The mass spectrometry data have been deposited in the ProteomeXchange Consortium via the MassIVE (Mass Spectrometry Interactive Virtual Environment) partner repository under the dataset identifier PXD062819. The transcriptome data are available upon request by email to the authors. The raw metagenome sequences for HBB and CRA samples are available in BioProject PRJNA999720 [57] in the NCBI BioProject database. [Correction added on 07.10, after first online publication: Dataset identifiers have been updated.]
