# Supplementary material for: Omics Insights Into the Effects of Highbush Blueberry and Cranberry Crop Agroecosystems on Honey Bee Health and Physiology
Source: Proteomics. 2025 Sep 6;26(8):41–57. doi: 10.1002/pmic.70033 (PMC13419346; doi:10.1002/pmic.70033)
Supplement: Supplementary file 1 — Supporting File: pmic70033‐sup‐0001‐SuppMat.docx. [file PMIC-26--s001.docx]

**Supplementary Figures**

**
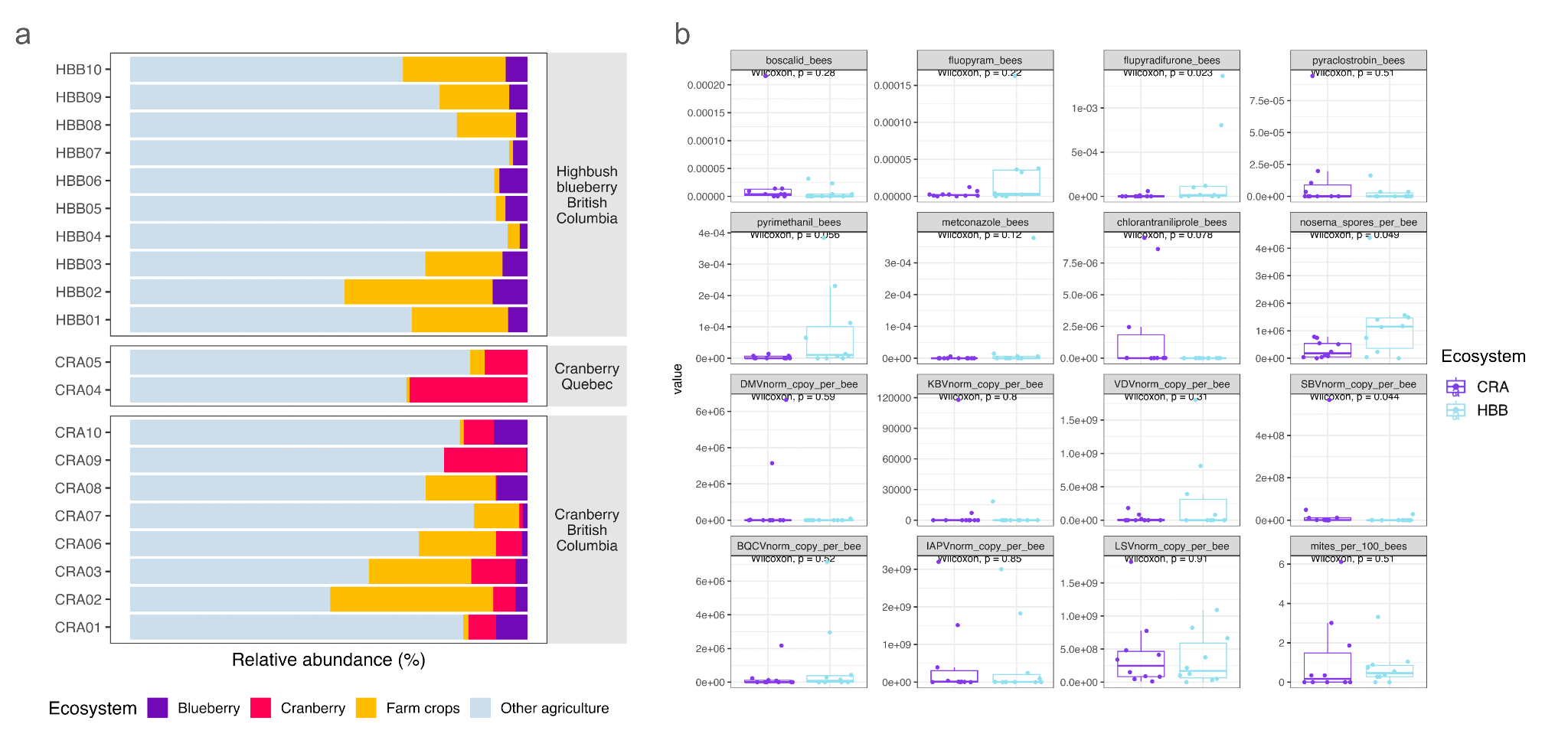
**

**Supplementary Figure 1.** **Visual description of sampling sites**

a. Relative abundance of land use types in Vancouver and Québec City

b. Box plot representation of pesticide and parasite counts per ecosystem


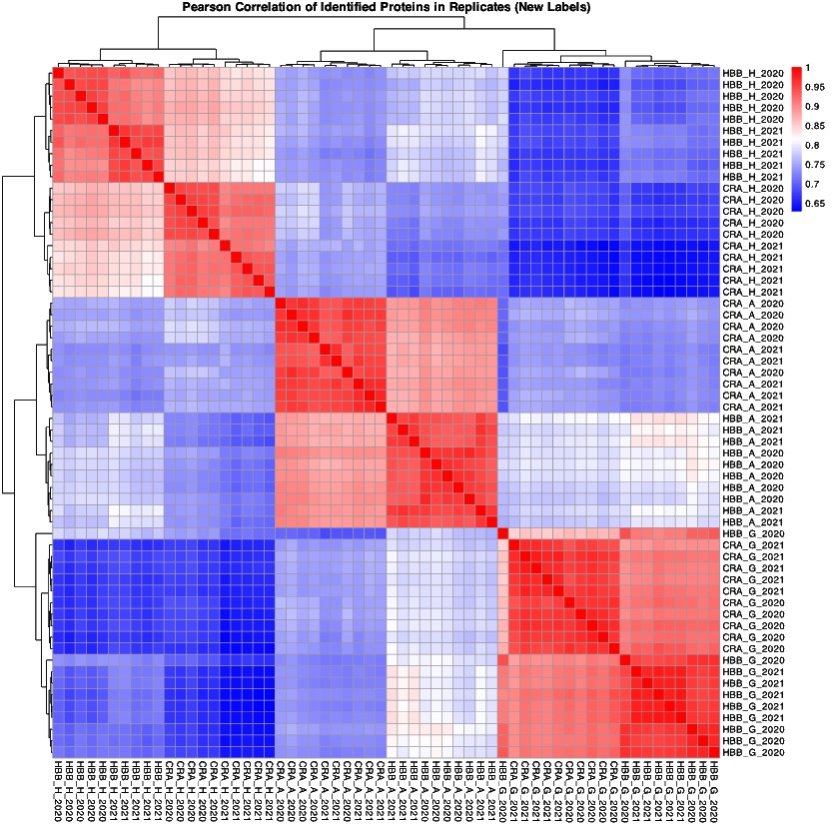


**Supplementary Figure 2.** **Pearson correlation heatmap of identified proteins across replicates in honey bees exposed to blueberry and cranberry ecosystems.**

The heatmap represents the Pearson correlation coefficients of proteomic profiles between biological replicates, with hierarchical clustering applied to identify similarity patterns across different groups. The color scale indicates correlation strength, where red denotes high correlation (close to 1.0) and blue indicates low correlation (close to 0.65). Labels on both axes correspond to individual replicate samples from different tissues (H = head, A = abdomen, and G = gut) and years (2020, 2021).


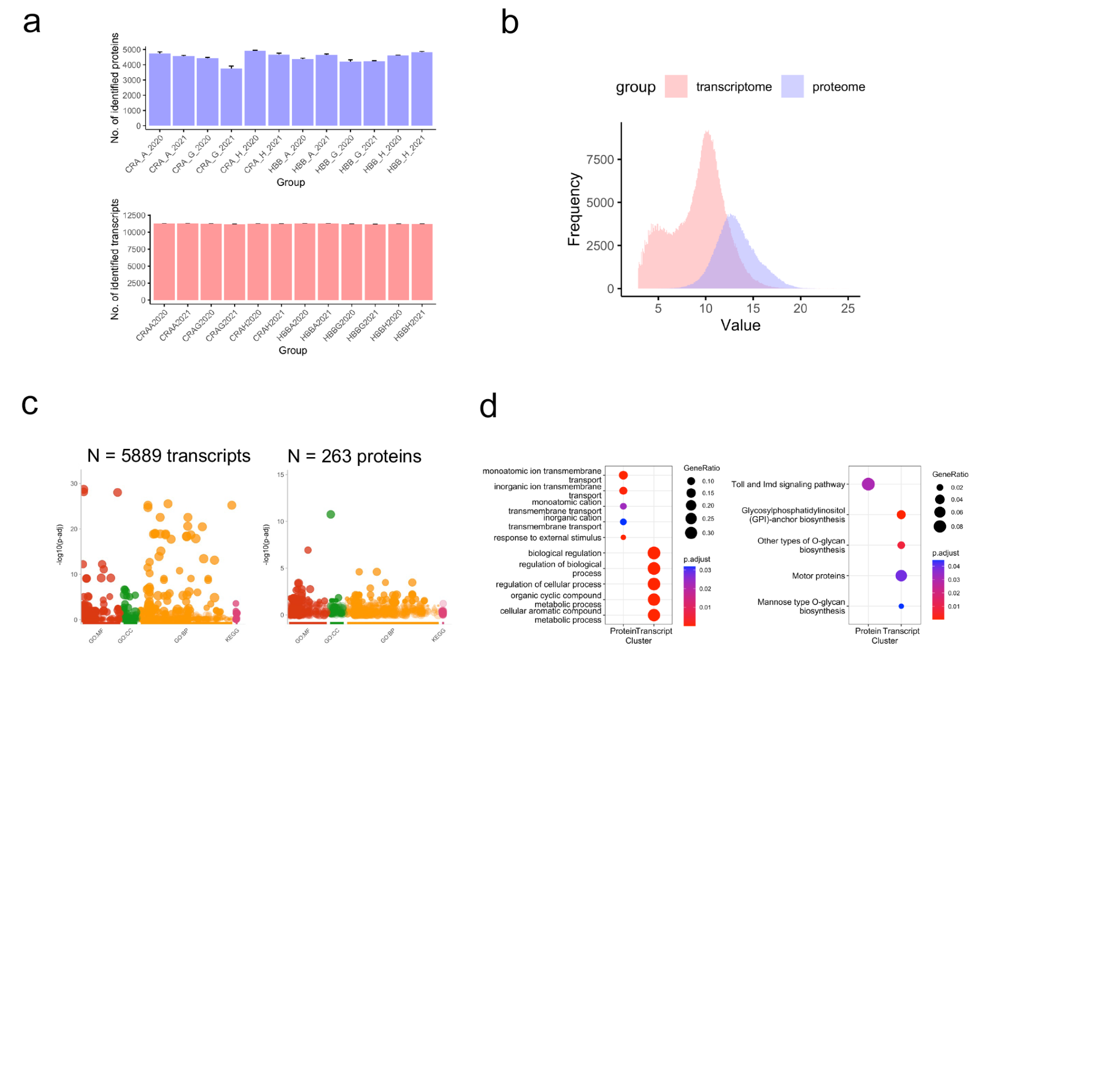


**Supplementary Figure 3.** **The proteomic and transcriptomic atlases of bees**

a. Numbers of identified proteins in LC–MS/MS proteomics and the number of genes in RNA-seq analysis of all samples.

b. Distributions of the bee proteomic (purple) and transcriptomic (red) landscapes based on the mean values of estimated copy number values across all the samples.

c. Scatterplots depict the distribution of functional enrichment analysis in transcripts (left, *n* = 5,889) and proteins (right, *n* = 263). Each dot represents the terms including GO term and KEGG pathways that were enriched exclusively at the transcriptomic or proteomic level, respectively.

d. Representative GO/KEGG functional annotations for genes from panel c. One-sided Fisher’s exact test, *p* value < 0.05. The dot size represents the number of proteins/transcripts involved in the relevant term. The color bar indicates the enrichment significance.

**Supplementary Figure 4.** **Tissue-specific differential protein expression and functional enrichment analysis in honey bees exposed to blueberry and cranberry ecosystems.**

a. PCA showing the tissue- and ecosystem-specific patterns of honey bee proteomes.

Each point represents the proteome of one honey bee tissue sample. Points are color coded according to their tissue (A = abdomen, G = gut, H = head) and ecosystem (HBB = highbush blueberry, CRA = cranberry).
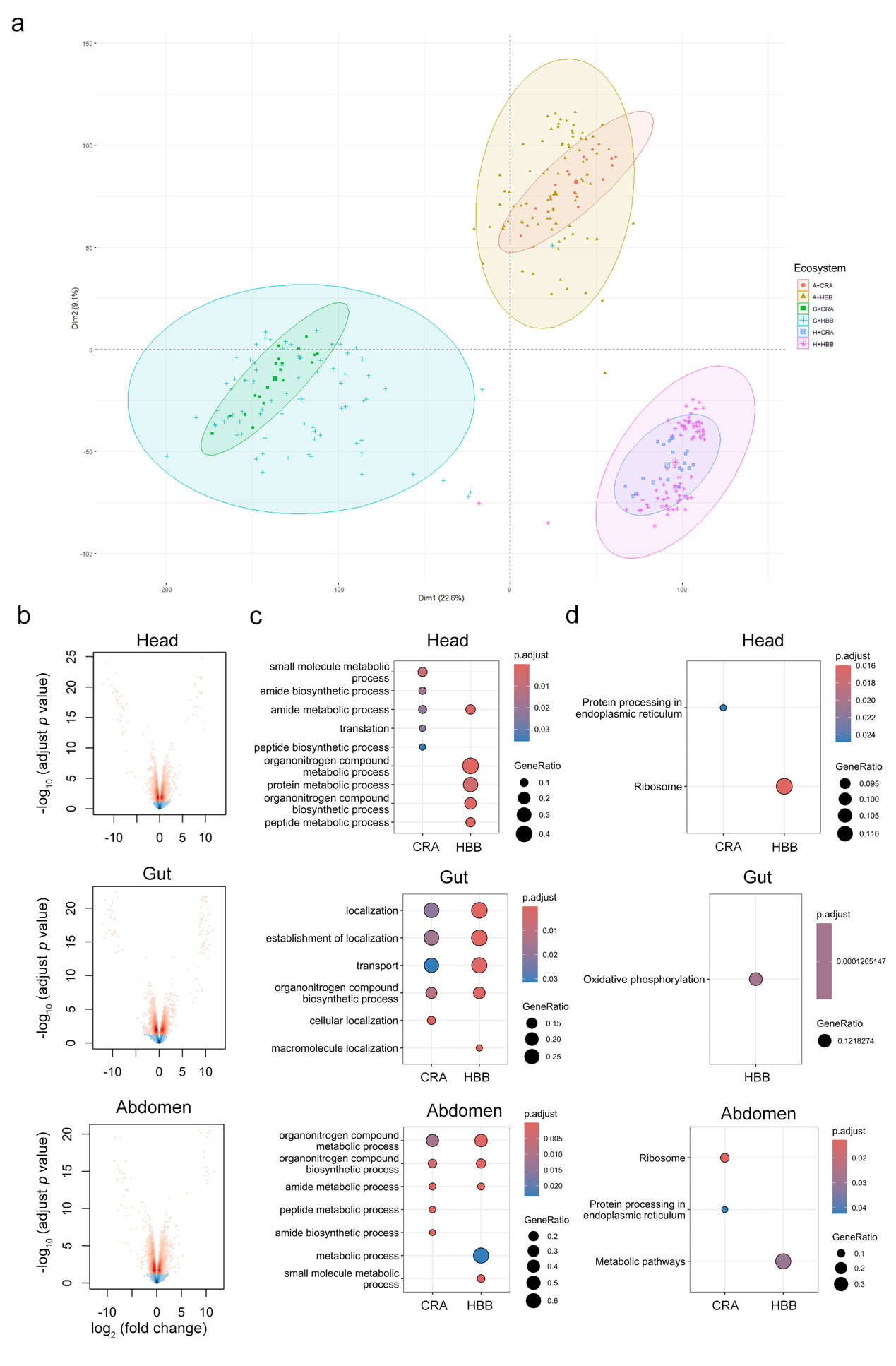


b-d. This presents tissue-specific proteomic responses in the head (top), gut (middle), and abdomen (bottom) of honey bees, highlighting differentially expressed proteins and their associated biological pathways.

b. Volcano plots depicting the log_2_ fold change (x-axis) and statistical significance (-log_10_ FDR, y-axis) of differentially expressed proteins. Each dot represents a protein, with proteins upregulated in the blueberry ecosystem (red) and those upregulated in the cranberry ecosystem (blue) highlighted.

c. GO term enrichment analysis for biological processes associated with differentially expressed proteins. Dots represent enriched functional categories, with dot size indicating gene ratio (proportion of genes in a category) and color indicating adjusted *p* value, where darker red signifies higher significance. Biological processes upregulated in the HBB and those upregulated in the CRA are shown separately.

d. KEGG pathway enrichment analysis, showing major metabolic pathways affected in each tissue. Pathways upregulated in the HBB and those upregulated in the CRA are shown separately. Dot size indicates gene ratio, and color represents the adjusted *p* value significance.


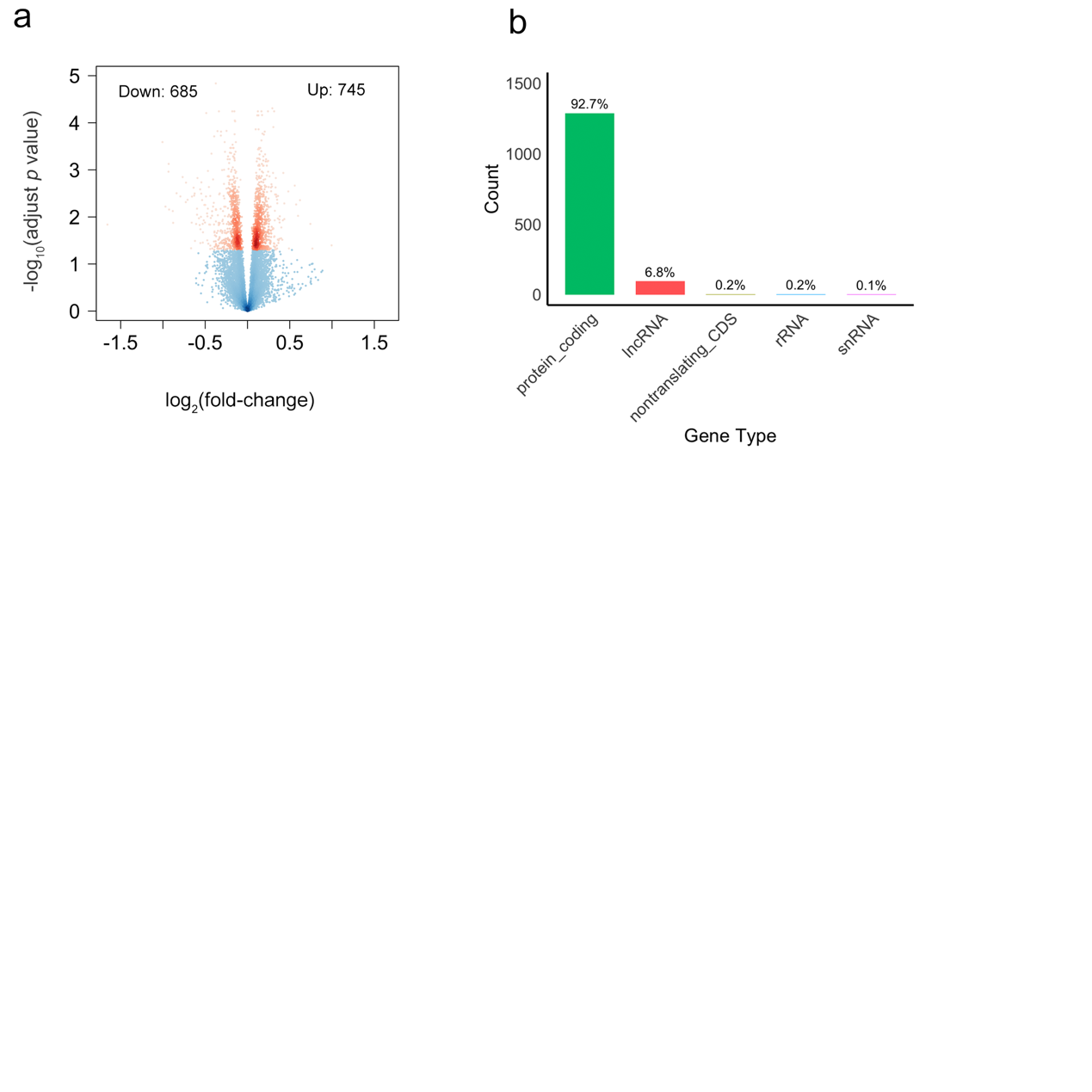


**Supplementary Figure 5. Differentially expressed transcripts and their gene type distributions.**

a. Volcano plot showing differential transcript expression between blueberry and cranberry ecosystems. Transcripts that were significantly expressed in the blueberry ecosystem are shown in red, while non-significant transcripts are shown in blue. The x-axis represents log₂ fold-change, and the y-axis represents -log₁₀ (adjusted *p* value).

b. Bar plot showing the distribution of significantly differentially expressed transcripts across gene types. Abbreviations: lncRNA, long non-coding RNA; CDS, coding DNA sequence (non-translating); rRNA, ribosomal RNA; snRNA, small nuclear RNA.


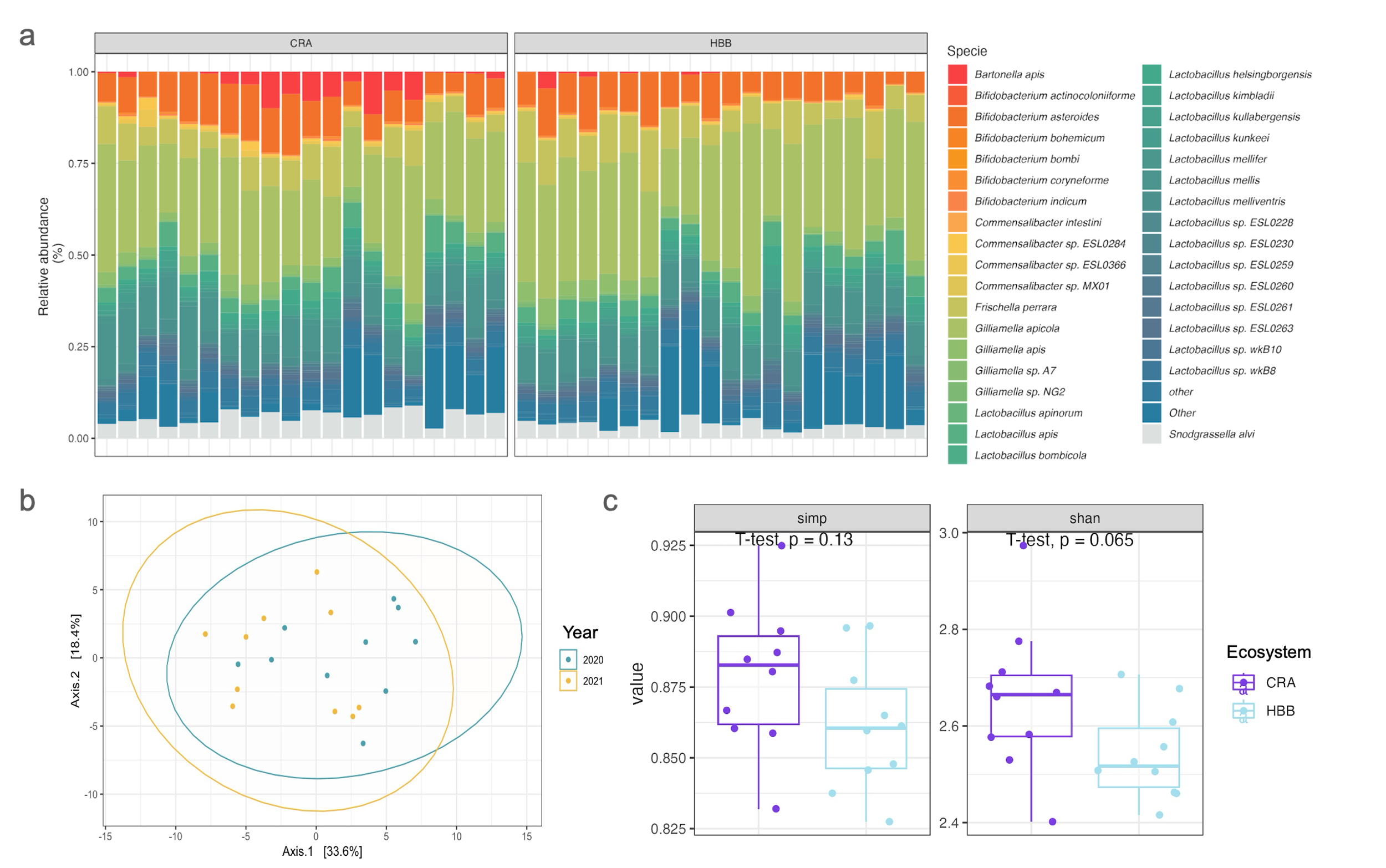


**Supplementary Figure 6.** **Relative abundance of gut microbiome composition in honey bees foraging in cranberry and blueberry ecosystems.**

a. Stacked bar plot represents the relative abundance (%) of bacterial species identified in the gut microbiome of honey bees collected from cranberry and blueberry pollination environments. Each vertical bar corresponds to an individual bee sample, with different colors representing distinct bacterial species as indicated in the legend on the right.

b. PCoA illustrates no differences between bacterial taxa from bee gut in different years.

c. Box-plots of alpha-diversity Simpson and Shannon coefficients based on microorganisms counts from bees’ gut, taken from different ecosystems.


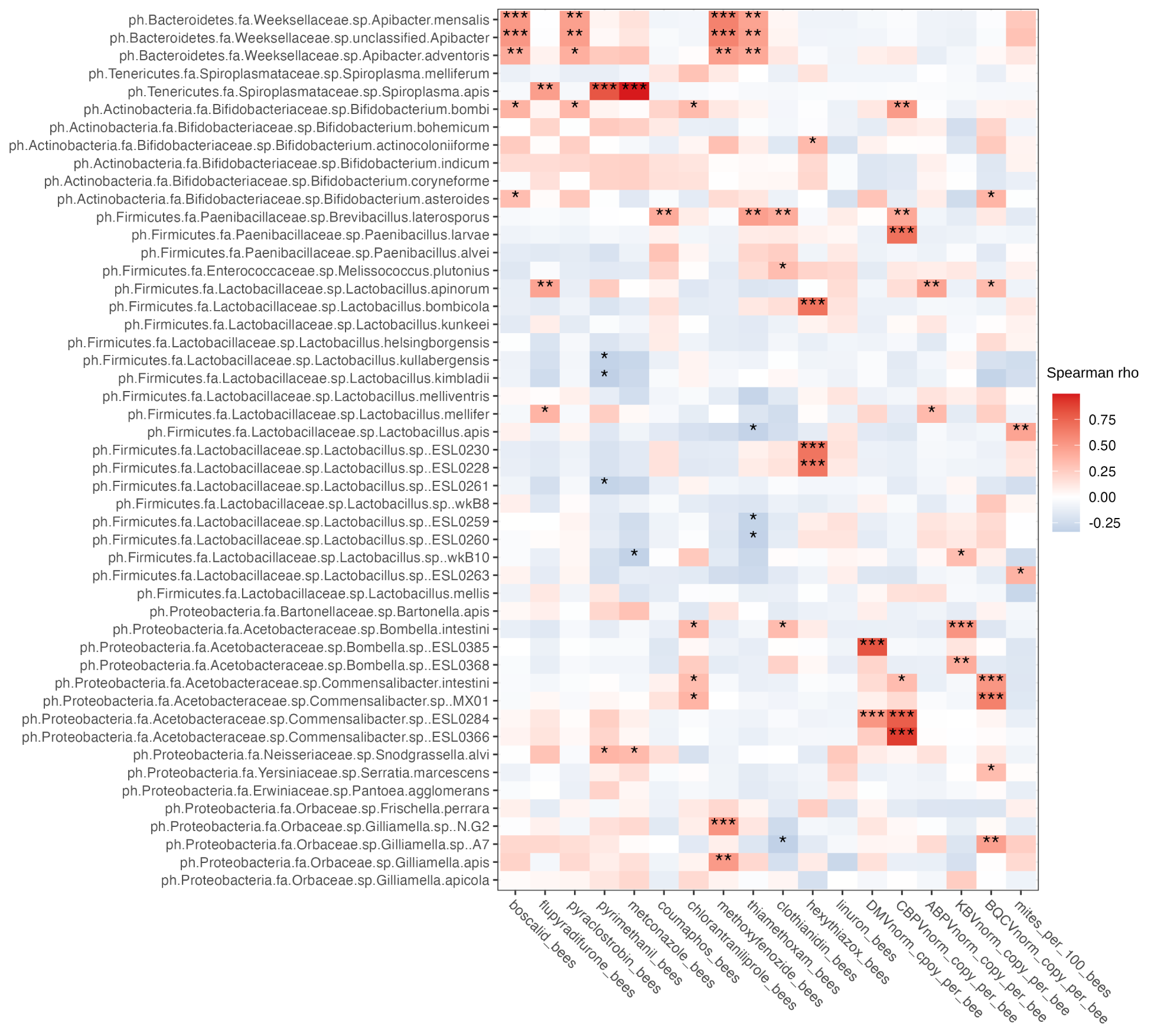


**Supplementary Figure 7.** **Association between gut microbiome composition and environmental stressors in honey bees.**

Heatmap illustrates the correlation between bacterial taxa (y-axis) and various environmental factors (x-axis), including pathogens, parasites, pesticide exposure, and other agroecosystem-specific stressors (**p* value < 0.05, ***p* value < 0.01, ****p* value < 0.001).
